# A Novel XRE-Type Regulator Mediates Phage Lytic Development and Multiple Host Metabolic Processes in Pseudomonas aeruginosa

**DOI:** 10.1128/spectrum.03511-22

**Published:** 2022-11-29

**Authors:** Xiang Long, Xiaolong Wang, Daqing Mao, Weihui Wu, Yi Luo

**Affiliations:** a College of Environmental Science and Engineering, Ministry of Education Key Laboratory of Pollution Processes and Environmental Criteria, Nankai University, Tianjin, China; b State Key Laboratory of Pollution Control and Resource Reuse, School of the Environment, Nanjing University, Nanjing, China; c School of Medicine, Nankai University, Tianjin, China; d State Key Laboratory of Medicinal Chemical Biology, Key Laboratory of Molecular Microbiology and Technology of the Ministry of Education, College of Life Sciences, Nankai University, Tianjin, China; Wuhan University

**Keywords:** *Pseudomonas aeruginosa*, XRE family transcriptional regulator, LfsT, phage infection, FA metabolism, SPD transport, T3SS, fatty acid metabolism, spermidine transport

## Abstract

Pseudomonas aeruginosa is a Gram-negative opportunistic pathogen, the leading cause of acute and chronic infections in immunocompromised patients, frequently with high morbidity and mortality rates. The xenobiotic response element (XRE) family proteins are the second most common transcriptional regulators (TRs) in P. aeruginosa. However, only a few XRE-like TRs have been reported to regulate multiple bacterial cellular processes, encompassing virulence, metabolism, antibiotic synthesis or resistance, stress responses, and phage infection, etc. Our understanding of what roles these XRE-like small regulatory proteins play in P. aeruginosa remains limited. Here, we aimed to decipher the role of a putative XRE-type transcriptional regulator (designated LfsT) from a prophage region on the chromosome of a clinical P. aeruginosa isolate, P8W. Southern blot and reverse transcription quantitative PCR (RT-qPCR) assays demonstrated that LfsT controlled host sensitivity to the phage PP9W2 and was essential for efficient phage replication. In addition, electrophoretic mobility shift assays (EMSAs) and transcriptional *lacZ* fusion analyses indicated that LfsT repressed the lysogenic development and promoted the lytic cycle of phage PP9W2 by binding to the promoter regions of the *gp71* gene (encoding a CI-like repressor) and several vital phage genes. Combined with RNA-seq and a series of phenotypic validation tests, our results showed that LfsT bound to the flexible palindromic sites within the promoters upstream of several genes in the bacterial genome, regulating fatty acid (FA) metabolism, spermidine (SPD) transport, as well as the type III secretion system (T3SS). Overall, this study reveals novel regulatory roles of LfsT in P. aeruginosa, improving our understanding of the molecular mechanisms behind bacterium-phage interactions.

**IMPORTANCE** This work elucidates the novel roles of a putative XRE family TR, LfsT, in the intricate regulatory systems of P. aeruginosa. We found that LfsT bound directly to the core promoter regions upstream of the start codons of numerous genes involved in various processes, including phage infection, FA metabolism, SPD transport, and the T3SS, regulating as the repressor or activator. The identified partial palindromic motif NAACN(5,8)GTTN recognized by LfsT suggests extensive effects of LfsT on gene expression by maintaining preferential binding to nucleotide sites under evolutionary pressure. In summary, these findings indicate that LfsT enhances metabolic activity in P. aeruginosa, while it reduces host resistance to the phage. This study helps us better understand the coevolution of bacteria and phages (e.g., survival comes at a cost) and provides clues for designing novel antimicrobials against *P. aeruginosa* infections.

## INTRODUCTION

Phages have long been considered the predators of bacteria since 100 years ago. However, recent studies have indicated that bacterium-phage interactions are far more complex than initially thought (1). Bacterium-phage coevolution is a vital driver of many ecological and evolutionary processes such as population dynamics, the evolution of diversity, mutation rates, and evolutionary pathogen virulence (2–4). The phage-host arms race is maintained during phage adsorption, injection into bacteria, replication inside host cells, and prophage lysogeny (5). Bacterial defense mechanisms against phage predation can be broadly classified into two categories. The first category includes surface modifications that directly impair phage adsorption. The second category involves interference in DNA replication, transcription, and translation processes after the phage genome is injected into the bacteria, e.g., restriction-modification (RM) endonucleases, inhibitory proteins (Abi and Sie), bacteriophage exclusion (Brex), and CRISPR (6). Despite the many new types of defense systems that have been identified recently, including RNA editing, retron satellite DNA synthesis, chemical antiphage defense systems, and quorum sensing (7–9), our understanding of the underlying molecular mechanisms of bacterium-phage interactions remains limited. In addition, temperate phages decide between lytic (where they replicate and lyse their host) and lysogenic (where they integrate and keep the host viable) cycles during infections (10). Apart from the many intracellular signaling pathways and genetic circuits that contribute to this decision, communication between phages using small molecules also profoundly influences it (11). These interplays between bacteria and phages result in mutual benefits and progress rather than unilateral extinction.

Pseudomonas aeruginosa is the primary agent causing nosocomial infections of the lungs, wounds, blood, and urinary tract, often accompanied by high morbidity and mortality rates (12). Acute and chronic infections caused by this bacterium in critically ill patients are difficult to treat due to its adaptability and resistance to multiple antimicrobials (13). Moreover, P. aeruginosa is a versatile metabolic bacterium with intricate regulatory systems that help it survive in changing environments (14). Under different conditions, transcriptional regulation is instrumental in controlling gene expression for enduring diverse stresses (15). The xenobiotic response element (XRE) family transcriptional regulators (TRs) are the second most common TRs in P. aeruginosa, regulating the expression of various genes, encompassing virulence factor genes, metabolic genes, antibiotic synthesis and resistance genes, stress response genes, and phenotypic switching genes (16–21). Although the regulatory mechanism of XRE TRs induced by many xenobiotics (e.g., heavy metals, oxidants, toxins, and antibiotics) is well known in eukaryotes (22, 23), their performance in prokaryotes is still unclear. Furthermore, only a fraction of bacterial studies have focused on the XRE family TRs (24–27), so the functions of the XRE TRs in P. aeruginosa require further investigation.

Here, we analyze the mode of action of LfsT in phage PP9W2 infection and multiple metabolic processes of the bacterial strain P8W. Our results indicate that the putative XRE-type TR LfsT functions as a global regulator by binding directly to the core promoter regions of the *gp71* gene (encoding a CI-like repressor protein) and several essential phage genes (encoding helicases or structural proteins) during infection. Besides, it also binds to the promoters of individual genes and divergent operons in the host genome, playing a role in diverse metabolic pathways, including fatty acid (FA) degradation, spermidine (SPD) transport, and the type III secretion system (T3SS). Taken together, these results show that LfsT mediates efficient phage replication and various bacterial metabolic pathways, helping the host fine-tune gene regulation during adaptation to different environments.

## RESULTS

### A putative regulator, LfsT, controls the phage sensitivity of the host.

Phage PP9W2 (GenBank accession number OM141125) is a linear double-stranded DNA phage with a 54-kb genome (see Fig. S1A in the supplemental material), which was isolated in our previous study (28). Its major capsid protein is highly homologous to those of seven related D3-like Pseudomonas phages (using lipopolysaccharide [LPS] as a phage receptor) according to phylogenetic tree analysis (Fig. S1B). Growth inhibition experiments indicated that the best MOI (multiplicity of infection) for phage PP9W2 was 1 (Fig. S1C). Furthermore, a one-step growth experiment demonstrated that the latency time and the burst size of phage PP9W2 were about 1.5 to 2 h and 99, respectively (Fig. S1D). We constructed a Tn*5G* transposon (Table 1) insertion library of the clinical P. aeruginosa strain P8W (GenBank accession number NZ_CP081477.2) (sensitive to PP9W2) to screen for phage-resistant (PR) mutants. A total of five PR mutants (Fig. S2) were found, and four insertion sites (I1 to I4) were located at a cluster of genes associated with LPS synthesis in P. aeruginosa (29–31). Meanwhile, only one (I5) was inserted immediately behind bp 81 of a putative transcription factor gene (GenBank accession number WP_006379425.1) from a predicted prophage region of P8W (32), comprising the XRE family helix-turn-helix (XRE-HTH) DNA binding domain (Fig. S3A). For convenience, we use the gene name *lfsT* (designated by its functions in the lytic development of phage PP9W2 and in the fatty acid metabolism, spermidine transport, and the T3SS of the bacterium P8W) throughout this article. PR1 to -4 exhibited severely truncated structures containing few core components and lost the O-chain part of the LPS profile compared to PR5 and the parent strain P8W (Fig. S3B). Moreover, we found significantly decreased LPS contents and adsorption rates of PR1 to -4 compared to PR5 and P8W (Fig. S3C and D). The phage resistance of PR1 to -4 may be attributed to the ineffective arrest of the infection process. However, the underlying molecular mechanism of the phage resistance of PR5 has not yet been determined as it displayed an LPS profile and an adsorption rate similar to those of the wild type.

**TABLE 1:** Bacterial strains, phages, and plasmids used in this work

Strain, phage, or plasmid	Genotype and/or description	GenBank accession no., reference, or source
Strains		
P. aeruginosa		
P8W	Wild-type P. aeruginosa clinical isolate	NZ_CP081477.2
P8D	*lfsT* deletion derivative of P8W	This work
P8D/pCTX	Complemented (*lfsT*) strain of P8D	This work
PR1	Tn*5G* insertion mutant (*wapR*) of P8W	This work
PR2	Tn*5G* insertion mutant (*wapP*) of P8W	This work
PR3	Tn*5G* insertion mutant (*waaC*) of P8W	This work
PR4	Tn*5G* insertion mutant (*waaC*) of P8W	This work
PR5	Tn*5G* insertion mutant (*lfsT*) of P8W	This work
P8D/pUCP18	P8D carrying pUCP18	This work
P8W/pUCP18	P8W carrying pUCP18	This work
P8D/pCT72N	P8D carrying pUCP18::*gp72*	This work
P8D/pCT71N	P8D carrying pUCP18::*gp71*	This work
P8W/pCT72N	P8W carrying pUCP18::*gp72*	This work
P8W/pCT71N	P8W carrying pUCP18::*gp71*	This work
E. coli		
DH5α	F^−^ ϕ80 *lacZ*ΔM15 Δ(*lacZYA-argF*)*U169 endA1 recA1 endA1 hsdR17*(r_K_^−^ m_K_^+^) *supE44* λ^−^ *thi-1 gyrA96 relA1 phoA*	TianGen
S17-1	RP4-2 Tc::Mu Km::Tn*7* Tp^r^ Sm^r^ Pro Res^−^ Mod^+^	TransGen
JM109	*endA1 recA1 gyrA96 thi hsdR17*(r_K_^−^ m_K_^+^) *relA1 supE44D*(*lac*-*proAB*) [F′ *traD36 proAB laqI*^q^*Z*ΔM15]	Biomed
BL21(DE3)	F^−^ *ompT hsdS*_B_(r_B_^−^ m_B_^−^) *gal dcm*(DE3)	Biomed
DH5α/pET32a(+)	DH5α carrying pET32a(+)	This work
DH5α/pOEX	DH5α carrying pET32a(+)::*lfsT*	This work
Phages		
PP9W	Excised phage isolated previously	This work
PP9W2	Lyses P8W or is lysogenic in it	OM141125
PP27	Excised phage isolated previously	This work
Plasmids		
pRK2013Tn*5G*	Tn*5G*-carrying plasmid; Km^r^ Gm^r^	69
pEX18Tc	Gene replacement vector; Tc^r^ *oriT*^+^ *sacB*^+^	Weihui Wu
pEX18Tc::UD	*lfsT* gene knockout vector	This work
pUCP18	Broad-host-range shuttle vector; Ap^r^	Weihui Wu
pCTX	*lfsT* gene complementation vector	This work
pCT72N	pUCP18::*gp72*	This work
pCT71N	pUCP18::*gp71*	This work
pMD19 (Simple)	Linearized T vector for cloning promoter fragments; Ap^r^	TaKaRa
pCP75	*gp75* gene promoter cloned into pMD19	This work
pCP72	*gp72* gene promoter cloned into pMD19	This work
pCP71	*gp71* gene promoter cloned into pMD19	This work
pCP13	*gp13* gene promoter cloned into pMD19	This work
pCP09	*gp09* gene promoter cloned into pMD19	This work
pCP04	*gp04* gene promoter cloned in pMD19	This work
pCP01	*gp01* gene promoter cloned into pMD19	This work
pCPP	*potA* gene promoter cloned into pMD19	This work
pCPF	*faoA* gene promoter cloned into pMD19	This work
pCPN	*popN* gene promoter cloned into pMD19	This work
pCP2	*PA2550* gene promoter cloned into pMD19	This work
pDN19lacΩ	Promoterless *lacZ* fusion vector; Sp^r^ Sm^r^ Tc^r^	Weihui Wu
pDN75	*gp75* gene promoter cloned into pDN19lacΩ	This work
pDN71	*gp71* gene promoter cloned into pDN19lacΩ	This work
pDN13	*gp13* gene promoter cloned into pDN19lacΩ	This work
pDN09	*gp09* gene promoter cloned into pDN19lacΩ	This work
pDN04	*gp04* gene promoter cloned into pDN19lacΩ	This work
pDN01	*gp01* gene promoter cloned into pDN19lacΩ	This work
pDNP	*potA* gene promoter cloned into pDN19lacΩ	This work
pDNF	*faoA* gene promoter cloned into pDN19lacΩ	This work
pDNN	*popN* gene promoter cloned into pDN19lacΩ	This work
pDN2	*PA2550* gene promoter cloned into pDN19lacΩ	This work
pET32a(+)	Fusion vector for an N-terminal His tag; Ap^r^	Novagen
pOEX	*his*-*lfsT* fusion in the pET32a(+) vector; Ap^r^	This work
pEASY-T1	Cloning vector for plotting the standard curves; Ap^r^ Kan^r^	TransGen
pEASY-L	Partially integrated fragment in pEASY-T1	This work
pEASY-1789	Partial *PA1789* fragment in pEASY-T1	This work

**FIG 1:**
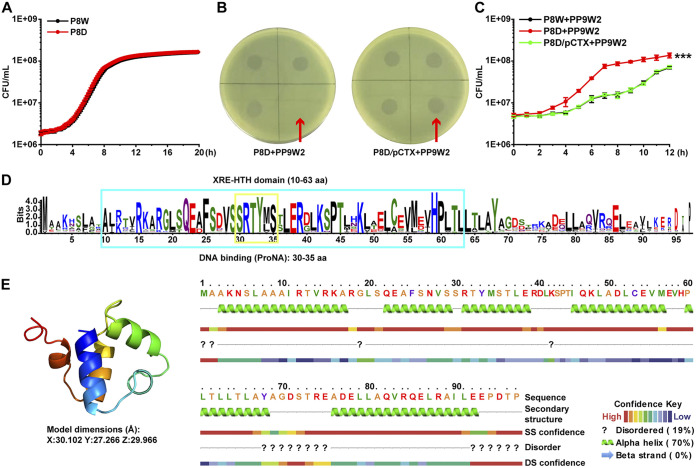
A hypothetical XRE-type transcriptional regulator, LfsT, impacts the sensitivity of bacterial strain P8W to phage PP9W2. (A) Growth experiment in LB medium for 12 h. The differently colored lines represent P8W and P8D (Δ*lfsT*). (B) Complementation (plasmid pCTX carrying the *lfsT* gene) restored host phage sensitivity in a spotting assay. (C) Growth inhibition of PP9W2 on the indicated strains (LB medium supplemented with 100 mg/L ampicillin) at an MOI (multiplicity of infection) of 1. The differently colored lines represent P8W, P8D, and P8D/pCTX. (D) Protein domain prediction for LfsT. The blue box represents an XRE-HTH domain from aa 10 to 63. The yellow box represents a DNA binding site from aa 30 to 35. More highly conserved amino acid residues are indicated by larger letters. (E, left) Protein structural model of LfsT built using SWISS-MODEL. The image is rainbow colored from the N to the C termini. (Right) Protein secondary structure of LfsT. The experiments were independently replicated three times, and each sample was tested in triplicate (A to C). Data were analyzed by one-way analysis of variance (ANOVA) with Tukey’s multiple-comparison test (α < 0.05) to examine the mean differences between the data groups. ***, *P < *0.001. Error bars show standard deviations. SS, secondary structure; DS, disorder structure.

The regulation of the sensitivity of bacteria to the phage is an evolved mechanism during continual bacterium-phage interactions (33). Research in this area will improve our understanding of their relationships. We focused on the *lfsT* gene since it seems irrelevant to phage receptor modification. To better explore the functions of *lfsT*, we constructed a Δ*lfsT* derivate (P8D) (Table 1) of strain P8W by homologous recombination (for details, see Materials and Methods) to exclude other interferences (e.g., polar effects), rather than using the PR5 mutant directly in subsequent experiments. Growth curve assays indicated that the *lfsT* gene is not essential for host cell growth since no growth retardation was found in the P8D group (Fig. 1A). The introduction of the *lfsT* gene (using the recombinant plasmid pUCP18::*lfsT* [designated pCTX]) (Table 1) into P8D restored its sensitivity to phage PP9W2 (Fig. 1B). Further growth inhibition experiments (MOI = 1) demonstrated that P8W and P8D/pCTX (complementation group) displayed distinct growth delays compared to P8D when incubated with PP9W2 after 12 h (*P < *0.001) (Fig. 1C). The LfsT protein (97 amino acids [aa]) contains an XRE-HTH domain (aa 10 to 63) with a predicted DNA binding site (aa 30 to 35) (Fig. 1D; Data Set S1), and the structural model of LfsT mainly included four α-helices with a λ repressor-like DNA binding domain belonging to the SinR family (Fig. 1E; Data Set S2). These data suggested that LfsT is a putative regulator of the clinical P. aeruginosa strain P8W, controlling the host’s sensitivity to the phage PP9W2.

### LfsT is essential for phage replication.

**FIG 2:**
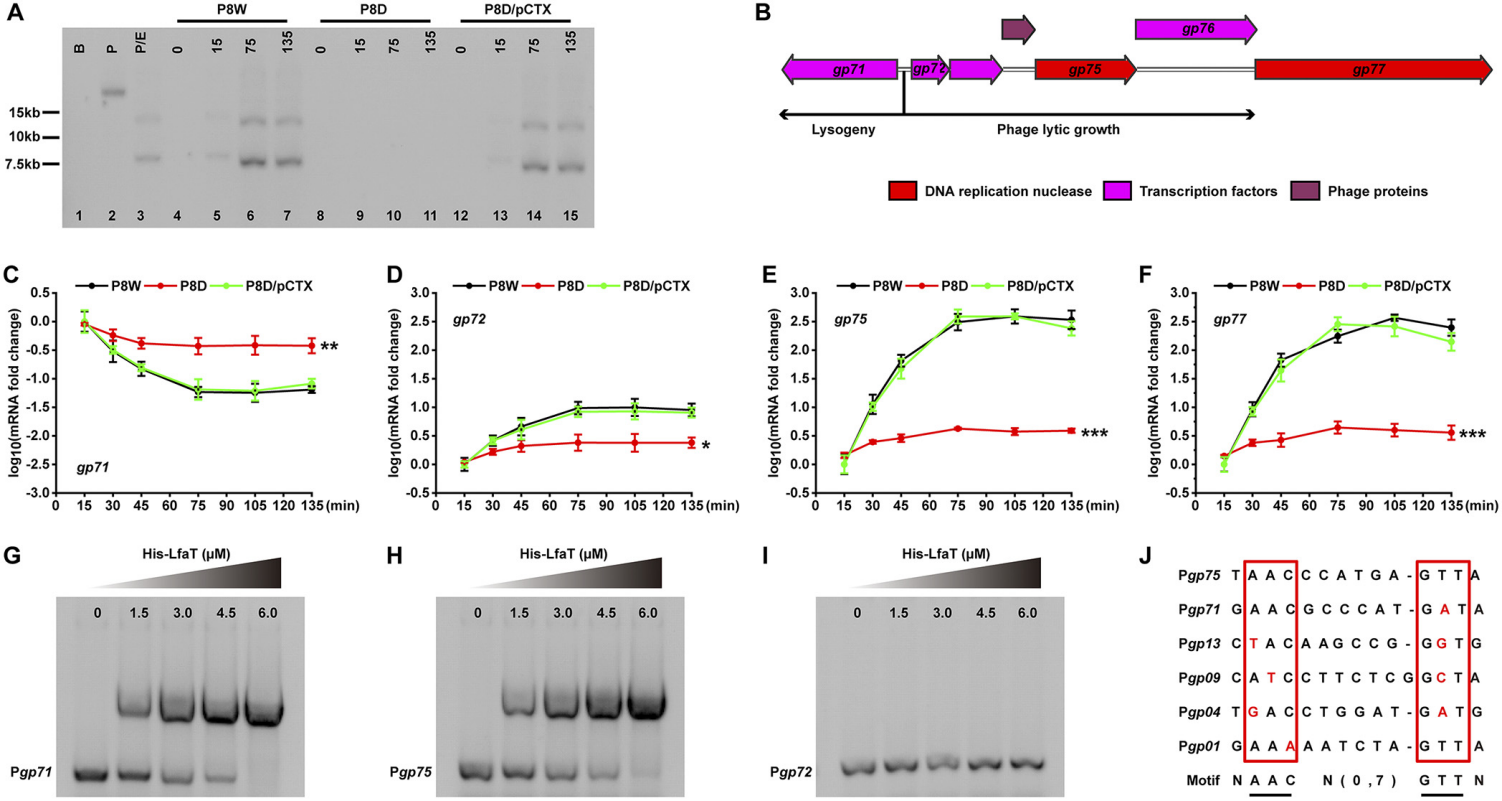
LfsT binds directly to the promoters of the *gp71* gene and other crucial phage genes to influence phage replication. (A) Southern blotting (detailed in Materials and Methods). The genomic DNA in all lanes was digested with FseI, except for lane 2. Lane 1 (B), P8W genomic DNA; lanes 2 and 3 (P and P/E), phage PP9W2 genomic DNA; lanes 4 to 15, total DNA of different bacterial cells infected with phage PP9W2 at the indicated time intervals (0, 15, 75, and 135 min); lanes 4 to 7, P8W cells; lanes 8 to 11, P8D cells; lanes 12 to 15, P8D/pCTX cells. (B) Information on genes flanking the *gp71*/*gp72* switch. The differently colored squares represent various proteins as indicated above. (C to F) RT-qPCR assay of the genes using cDNA of different strains infected with phage PP9W2 at the indicated time intervals. *gp71* encodes a CI-like repressor protein, *gp72* encodes a Cro-like repressor protein, *gp75* encodes the putative helicase DnaK, and *gp77* encodes the putative helicase DnaB. (G to I) EMSAs of different promoter fragments using the purified His-tagged LfsT protein. (G and H) LfsT binding to the *gp71* or *gp75* promoter; (I) Negative control. The purity of the His-tagged LfsT protein is about 97.9%. (J) Predicted LfsT binding sites of different promoters. *gp13* encodes the putative tail component, *gp09* encodes the phage gp6-like head-tail connector protein, *gp04* encodes the phage portal protein, and *gp01* encodes the terminase small subunit. A partial palindromic motif was speculated to be AACN(0,7)GTT. Red letters represent probable mutation sites. Each sample was tested in triplicate, and the experiments were independently replicated three times (C to F). Data were analyzed by one-way ANOVA with Tukey’s multiple-comparison test (α < 0.05) to examine the mean differences between the endpoints of data groups. Error bars show standard deviations. *, *P < *0.05; **, *P < *0.01; ***, *P < *0.001.

We used restriction endonuclease digestion (Table S1) to investigate the packaging mode of PP9W2 genomic DNA (gDNA) (34). As shown in Fig. S4A (lane 4), purified PP9W2 genomic DNA digested with FseI displayed two significant bands. After heat treatment, band A was replaced by two new positive bands (lane 6, bands B and C). This result was consistent with the illustration in Fig. S4B. The 13.2-kb fragment (A) was from the digested concatemeric DNA, while the 7.7-kb fragment (B) and the 5.5-kb fragment (C) were from the digested monomeric DNA. These data indicated that PP9W2 genomic DNA uses site-specific *cos*-type-like packaging. To identify whether the genome replication of PP9W2 was disrupted in P8D, Southern blot analysis was performed. Only fragments with the designed DNA probe (Fig. S4B) could be detected. No positive bands were detected in P8D, while P8W and P8D/pCTX displayed similar band profiles (Fig. 2A). These results demonstrated that LfsT is required for the genome replication of phage PP9W2 during infection.

Bacteriophage λ encodes two proteins that play opposing roles in phage growth. The λ repressor CI is required for lysogeny, while the Cro repressor turns off early gene transcription (35). We found a similar CI/Cro switch in the genome of PP9W2 (Fig. 2B; Fig. S5), which might control phage lysis-lysogeny decisions. To further investigate how LfsT impacts phage replication, reverse transcription quantitative PCR (RT-qPCR) assays using different primer pairs (Table 2) were conducted for the indicated strains. After 75 min of infection, the expression levels of *gp71* (encoding a CI-like protein), *gp72* (encoding a Cro-like protein), *gp75* (encoding the putative helicase DnaK), and *gp77* (encoding the putative helicase DnaB) reached the plateau phase in all of the tested strains (Fig. 2C to F). After another hour, the transcription levels of *gp72*, *gp75*, and *gp77* in P8D were significantly reduced compared to those in P8W and P8D/pCTX (*P < *0.05 and *P < *0.001) (Fig. 2D to F). Meanwhile, the transcription level of *gp71* in P8D displayed a contrasting increase (*P < *0.01) (Fig. 2C). These results suggested that LfsT may be a critical factor that influences the transcription of these phage genes.

**TABLE 2:** Primers used in this work

Primer name	Sequence (5′–3′)^*a*^	Function or description
OTn1	GATCCTGGAAAACGGGAAAG	Identification of Tn*5G* insertion sites
OTn2	CCATCTCATCAGAGGGTAGT	

lfsTU-F	CCGGAATTCCGGTCAGTCATGGCGAAAC	1-kb fragment upstream of *lfsT*
lfsTU-R	CGCGGATCCAACCAAGATGACAGCCCATTG	

lfsTD-F	CGCGGATCCGTTGGCGTGTGGTACTGAAT	1-kb fragment downstream of *lfsT*
lfsTD-R	CCCAAGCTTGCGTCCTTGTCTTGTTGTGA	

lfsT-F	CCGGAATTCGTGGCAGCGAAGAACTCATTG	Cloning of the *lfsT* gene into pUCP18
lfsT-R	CGCGGATCCCTAAGGCGTGTCGGGCTCTT	

72N-F	CCGGAATTCGGAAGCTATGACCACCATCTAC	Cloning of the *gp72* gene into pUCP18
72N-R	CGCGGATCCGCTGTCATGTCAGGCAACC	

71N-F	CCGGAATTCTTCGGATGGAACTCAAAGAC	Cloning of the *gp71* gene into pUCP18
71N-R	CGCGGATCCGCGATCTCCTATCCAATGC	

OEX-F	CGCGGATCCGTGGCAGCGAAGAACTCATTG	Expression of *lfsT* in pET32a(+)
OEX-R	CCCAAGCTTCTAAGGCGTGTCGGGCTCTT	

gp02-F^*b*^	AATTACGGAGTGTCGGTCTTC	DNA probe synthesis for Southern blotting
gp02-R	GGATGGATGTGTTGGAGAGTC	

gp71-F	AGCATGGAGCCTTACATATTCG	RT-qPCR assay for *gp71*
gp71-R	ATAAGCGGACTTGTCTGGATTG	

gp72-F	TTTGGGACTCAAGACGAGACC	RT-qPCR assay for *gp72*
gp72-R	CAACCGCCGACAGCATTTC	

gp75-F	GCGATTATTACCTGTCCGTAGA	RT-qPCR assay for *gp75*
gp75-R	GGTGTTCAGTCGGCAGATG	

gp77-F	GCGTTCCTGTGATTCTCCTAAG	RT-qPCR assay for *gp77*
gp77-R	ACTCGGTATGCTCGTTGTAGA	

gp58-F	AGCAGCGTAGTGATGAATGGT	RT-qPCR assay for *gp58*
gp58-R	AATCGGCTCCAGGTCGGTA	

gp36-F	CCACACTAAGGCAGGCAAG	RT-qPCR assay for *gp36*
gp36-R	CGCAGGTCGTGAATCGTAA	

faoA-F	GCAAGCCGAAGAAGGTCAC	RT-qPCR assay for *faoA*
faoA-R	GGAAGCCGATGCCGTAGAT	

2550-F	GCGGCAACATAGACCACATC	RT-qPCR assay for *PA2550*
2550-R	TGCATGGCGTACCAGTAGG	

potA-F	CAGCCTGACGATCAACACC	RT-qPCR assay for *potA*
potA-R	GCTCTGGAACACCGTATGC	

popN-F	GGACATCCTCCAGAGTTCCTC	RT-qPCR assay for *popN*
popN-R	AAGGCGAAGGTCAGCTCTT	

rpoD-F	CGTCCTCAGCGGCTATATCG	Reference gene for RT-qPCR assay
rpoD-R	TCTTCCTCGTCGTCCTTCTCT	

E71-F	ATGGGAACGCCCATGATAA	Promoter region of *gp71* for EMSA
E71-R	CTGAGCAATATACGCCGATC	

E72-F	CCCTGCTGTACCGTATGAGT	Promoter region of *gp72* for EMSA
E72-R	GACACAGGTCCTCTTTCTTGAA	
E75-F	GCGATTACTACAGGGCTTTGT	Promoter region of *gp75* for EMSA
E75-R	GTTCTGTCATGCCGATCTTGT	

E13-F	GGACAGCCTGGAGCACATT	Promoter region of *gp13* for EMSA
E13-R	CGCACCATCCGATCAAACC	

E09-F	TCTGCGGTGAGCTTCTGAC	Promoter region of *gp09* for EMSA
E09-R	ACCATGCCAGTGAGACATCC	

E04-F	TCGTGGACTTCAACCAGACA	Promoter region of *gp04* for EMSA
E04-R	TTTCCTTGGCGTCGATCTTTG	

E01-F	GAAATAGTCGGGTTCCATCAGC	Promoter region of *gp01* for EMSA
E01-R	GGTGTCCTAGCGAAAGGTTCT	

Epot-F	GGCGAAGGAACATCGAAGAC	Promoter region of *potA* for EMSA
Epot-R	CGCATCCCGCTCTAACTAGA	

Efao-F	GGCGTATGAATCGAGCGTTT	Promoter region of *faoA* for EMSA
Efao-R	CAAGAGGCTTAACCGTGATGG	

Epop-F	GCGACGAATTTCAGTGCCA	Promoter region of *popN* for EMSA
Epop-R	CGGAGGAACTCTGGAGGATG	

E255-F	TCAGGTTGGCTTCGGTATAGAT	Promoter region of *PA2550* for EMSA
E255-R	TGGTAACGATGCCGGAACA	

M13-47	CGCCAGGGTTTTCCCAGTCACGAC	Amplification of the promoter regions cloned into pMD19
RV-M	GAGCGGATAACAATTTCACACAGG	

L71-F	CCGGAATTCATGGGAACGCCCATGATAA	Promoter region of *gp71* for *lacZ* fusion
L71-R	CGCGGATCCCTGAGCAATATACGCCGATC	

L75-F	CCGGAATTCGCGATTACTACAGGGCTTTGT	Promoter region of *gp75* for *lacZ* fusion
L75-R	CGCGGATCCGTTCTGTCATGCCGATCTTGT	

L13-F	CCGGAATTCGGACAGCCTGGAGCACATT	Promoter region of *gp13* for *lacZ* fusion
L13-R	CGCGGATCCCGCACCATCCGATCAAACC	

L09-F	CCGGAATTCTCTGCGGTGAGCTTCTGAC	Promoter region of *gp09* for *lacZ* fusion
L09-R	CGCGGATCCACCATGCCAGTGAGACATCC	

L04-F	CCGGAATTCTCGTGGACTTCAACCAGACA	Promoter region of *gp04* for *lacZ* fusion
L04-R	CGCGGATCCTTTCCTTGGCGTCGATCTTTG	

L01-F	CCGGAATTCGAAATAGTCGGGTTCCATCAGC	Promoter region of *gp01* for *lacZ* fusion
L01-R	CGCGGATCCGGTGTCCTAGCGAAAGGTTCT	

Lpot-F	CCGGAATTCGGCGAAGGAACATCGAAGAC	Promoter region of *potA* for *lacZ* fusion
Lpot-R	CGCGGATCCCGCATCCCGCTCTAACTAGA	

Lfao-F	CCGGAATTCGGCGTATGAATCGAGCGTTT	Promoter region of *faoA* for *lacZ* fusion
Lfao-R	CGCGGATCCCAAGAGGCTTAACCGTGATGG	

Lpop-F	CCGGAATTCGCGACGAATTTCAGTGCCA	Promoter region of *popN* for *lacZ* fusion
Lpop-R	CGCGGATCCCGGAGGAACTCTGGAGGATG	

L255-F	CCGGAATTCTCAGGTTGGCTTCGGTATAGAT	Promoter region of *PA2550* for *lacZ* fusion
L255-R	CGCGGATCCTGGTAACGATGCCGGAACA	

LC-F	AGCCACAATCCTGTGCTCTAC	Quantification of lysogenic copies
LC-R	AAAGGAATTTCACGATTGGCAC	

TC-F^*c*^	AGGAAGGCTACAGCGTCTC	Quantification of total copies
TC-R	GGCGGTCTTGGTCATCAGT	

a The underlined sequences represent the sites of recognition of different restriction enzymes.

b Primers gp02-F and gp02-R were used to amplify the designed probe region of *gp02* (502 bp).

c Primers TC-F and TC-R were used to amplify the specific fragment of phage PP9W2 integrated into the genome of P8W.

Considering the performance of LfsT during phage infection and its predicted structure, we hypothesized that it might bind to the promoters of some phage genes and verified this by an electrophoretic mobility shift assay (EMSA). As shown in Fig. 2G and H, the purified His-tagged LfsT protein bound specifically to the promoters of *gp71* and *gp75*, and the promoter of *gp72* was used as the control group (Fig. 2H; Fig. S5). Combined with the other four promoter sequences bound by LfsT (Table S2), we predicted a flexible palindromic structure, NAACN(0,7)GTTN (Fig. 2J), in these potential binding sequences using an online tool, MEME Suite (36). These data indicated that LfsT probably regulates the expression of some related phage genes by binding directly to their promoters during infection.

### LfsT promotes phage lytic growth rather than lysogenic development.

**FIG 3:**
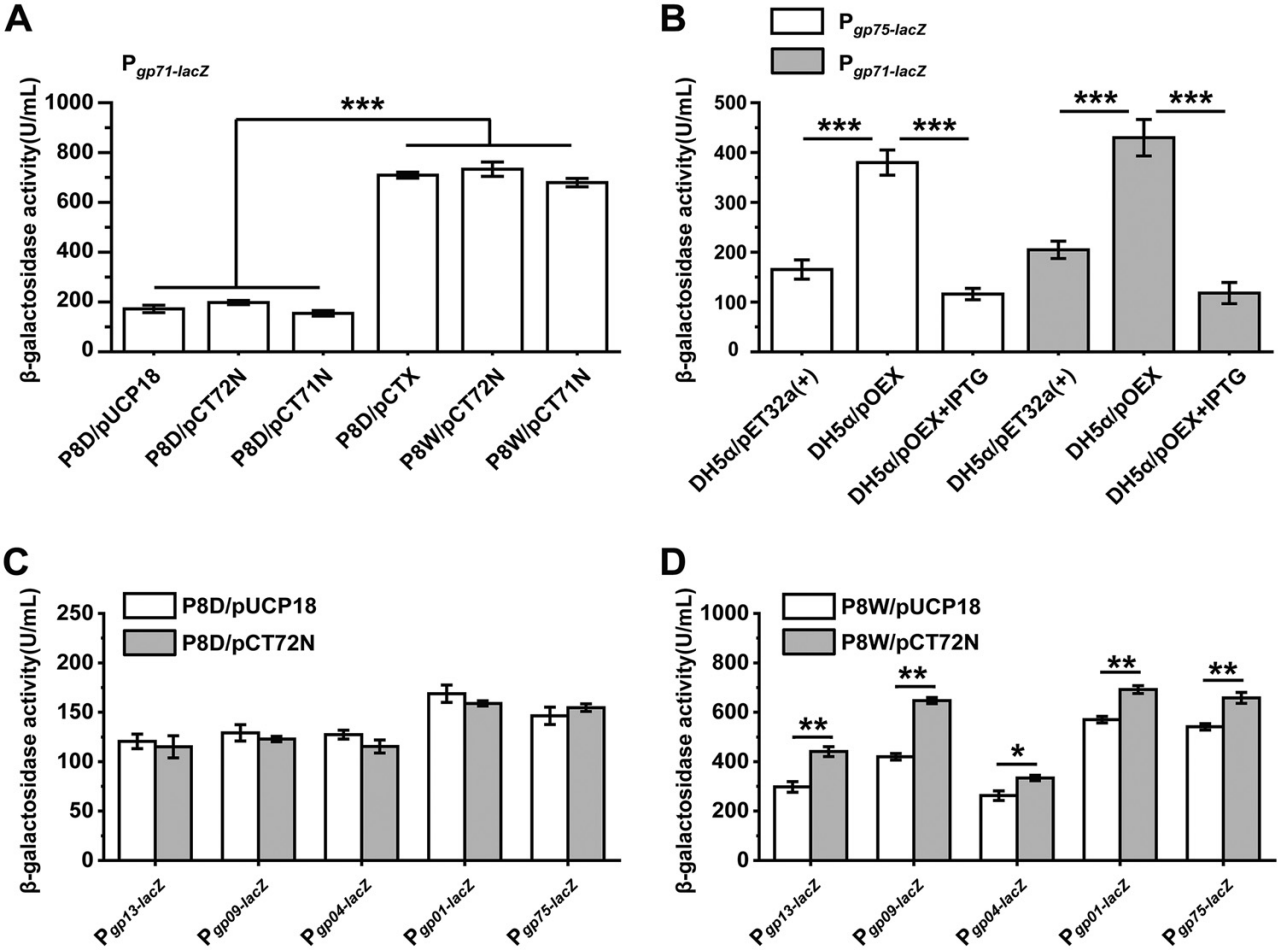
Function of LfsT in regulating vital phage genes. (A) LacZ activity in the indicated double transformants carrying pDN19lacΩ::P*gp71* (P*gp71*-*lacZ*) evaluated by the corresponding β-galactosidase activities. pUCP18, empty vector; pCT72N, pUCP18::*gp72*; pCT71N, pUCP18::*gp71*; pCTX, pUCP18::*lfsT*. (B) LacZ activity in the indicated E. coli DH5α double transformants carrying pDN19lacΩ::P*gp75* (P*gp75*-*lacZ*) or pDN19lacΩ::P*gp71* (P*gp71*-*lacZ*). pET32a(+) is an empty expression vector. pOEX, pET32a(+)::*lfsT*. The overexpression of *lfsT* was started by the addition of 1.5 mM isopropyl β-d-1-thiogalactopyranoside (IPTG). (C) LacZ activity of different phage gene promoter-*lacZ* fusions in P8D carrying pUCP18 or pCT72N. (D) LacZ activity corresponding to the results in panel C for P8W carrying pUCP18 or pCT72N. All of the double transformants were grown in LB medium supplemented with 100 mg/L ampicillin, streptomycin, and tetracycline. The experiments were independently replicated three times, and each sample was tested in triplicate. Data were analyzed by ANOVA with Tukey’s multiple-comparison test (α < 0.05) to examine the mean differences between the data groups. *, *P < *0.05; **, *P < *0.01; ***, *P < *0.001. Error bars show standard deviations.

To further explore the underlying regulatory mechanism of LfsT, we measured the β-galactosidase activities of various double transformants (15). Using a transcriptional *lacZ* reporter fusion, we found that the *gp71* promoter was more active in P8D/pCTX, P8W/pCT72N (pUCP18::*gp72*) (Table 1), and P8W/pCT71N (with the *lfsT* gene) than in P8D/pUCP18 (empty vector), P8D/pCT72N, and P8D/pCT71N (without the *lfsT* gene) (*P < *0.001) (Fig. 3A). Whether in the presence or the absence of *lfsT*, the complementation of the Gp71 or Gp72 protein did not affect LacZ activity in the corresponding cell extracts. These results hinted that LfsT probably binds directly to the promoter region of *gp71* to regulate its expression in P8W. Moreover, the CI/Cro-like Gp71/Gp72 switch in PP9W2 may be defective (Fig. S5). To further study the dose-effect patterns of LfsT (37), we overexpressed the *lfsT* gene in the heterologous host Escherichia coli DH5α treated with isopropyl β-d-1-thiogalactopyranoside (IPTG). Both the *gp75* and *gp72* promoters were significantly less active in response to LfsT (*P < *0.001) (Fig. 3B). These data suggested the stimulatory (i.e., low dose) and inhibitory (i.e., high dose) roles of LfsT in regulating the transcription of these phage genes, which is analogous to hormetic dose-response mechanisms. Besides, the expression levels of several phage genes (encoding structural proteins) bound by LfsT (Fig. 2J) were analyzed, including the putative tail component gene *gp13*, the phage gp6-like head-tail connector protein gene *gp09*, the phage portal protein gene *gp04*, the terminase small-subunit gene *gp01*, and the putative helicase DnaK gene *gp75*. The expression of all of these genes is unaffected by the Gp72 protein since no difference between the P8D/pUCP18 and P8D/pCT72N groups was observed (Fig. 3C). However, it was significantly enhanced to different extents in the presence of LfsT, by 48.1% (*P < *0.01), 54.2% (*P < *0.01), 27.2% (*P < *0.05), 21.3% (*P < *0.01), and 21.6% (*P < *0.01), respectively (Fig. 3D). These results indicated that the Gp72 protein is likely incapable of binding to the defective O_R_3-like operator and that the efficient transcription of these phage genes is dependent on LfsT.

**FIG 4:**
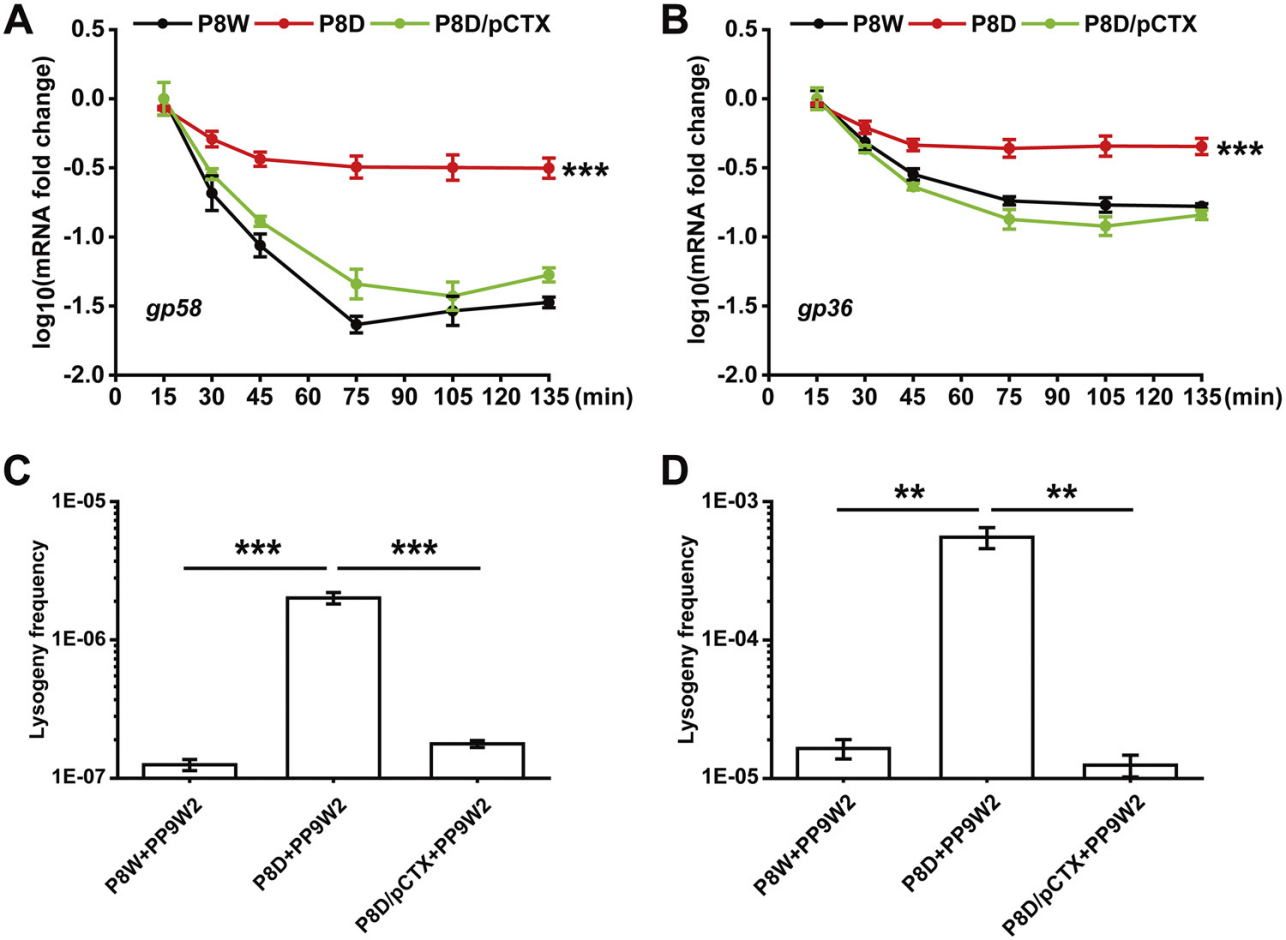
Negative effects of LfsT on lysogenic phage development. (A and B) RT-qPCR assay for *gp58* (encoding a YqaJ viral recombinase family protein) and *gp36* (encoding a phage integrase) in different strains (colored lines) infected with phage PP9W2 at the indicated time intervals. (C and D) Lysogeny frequencies of phage PP9W2 in the indicated strains after 2 h (C) or 12 h (D) of growth. The experiments were independently replicated three times, and each sample was tested in triplicate. Data were analyzed by one-way ANOVA with Tukey’s multiple-comparison test (α < 0.05) to examine the mean differences between the indicated groups. **, *P < *0.01; ***, *P < *0.001. Error bars show standard deviations.

Considering that LfsT might bind to a truncated O_R_3-like position adjacent to the −10 promoter region (Fig. S5), we then asked whether LfsT would interfere with phage lysogenic development. First, we tested the expression of several lysogeny-related genes in the indicated strains by RT-qPCR analysis. The expression levels of the *gp58* (encoding a YqaJ viral recombinase family protein) and *gp36* (encoding a phage integrase) genes in P8D were significantly increased in comparison to the levels in P8W or P8D/pCTX after 135 min of infection (*P < *0.001) (Fig. 4A and B). We also designed specific primer pairs (Table 2) to further calculate the related phage lysogeny frequencies by a quantitative PCR method (for details, see Materials and Methods). Although the lysogeny frequency of PP9W2 in all of the indicated strains increased with time (from 2 h to 12 h), it was significantly higher in P8D than in P8W or P8D/pCTX after 2 h (*P < *0.001) (Fig. 4C) or 12 h (*P < *0.01) (Fig. 4D) of growth. These data demonstrated that LfsT inhibits phage lysogenic development to a specific extent.

### Identification of LfsT binding sites in the genome of P8W.

**FIG 5:**
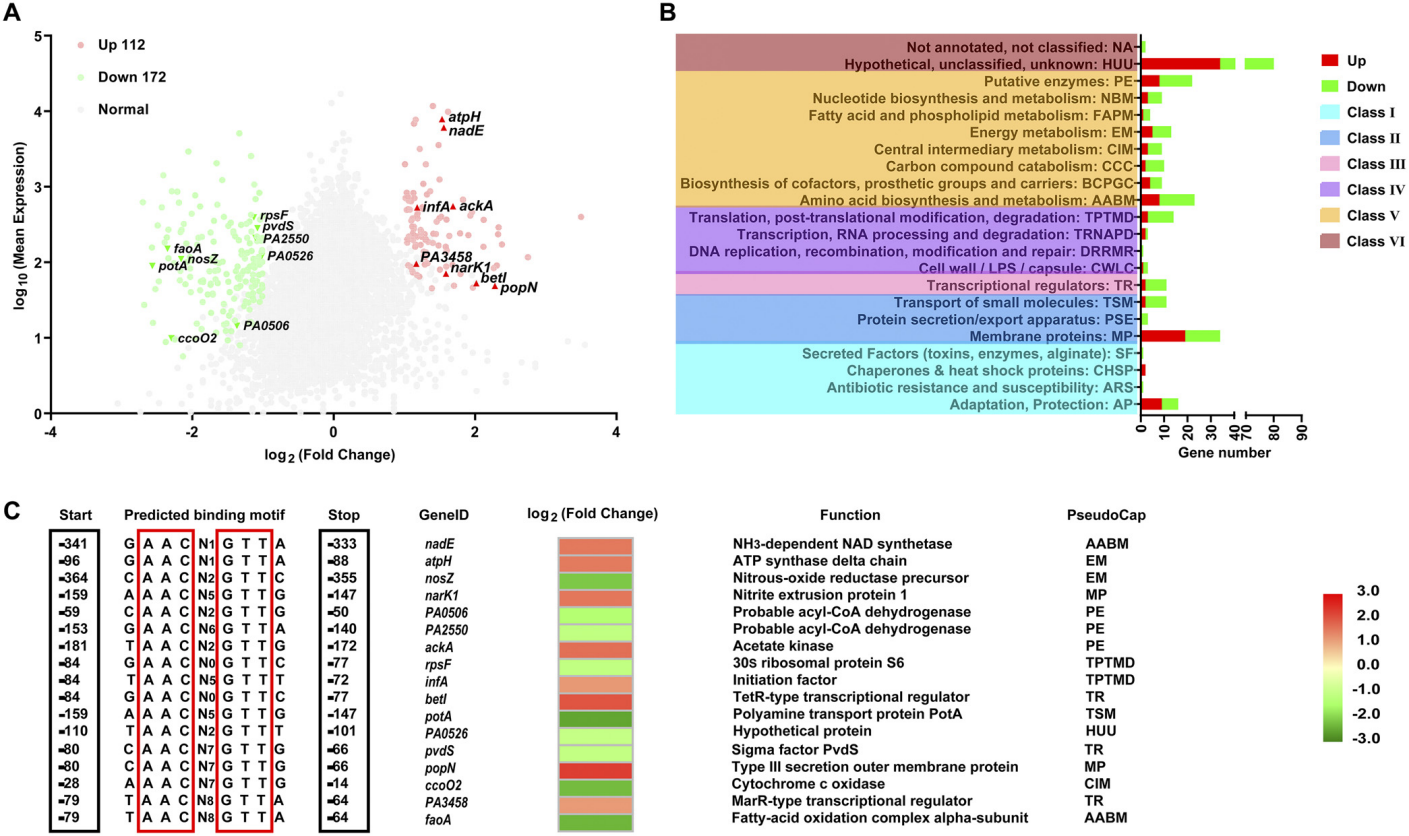
Predicted LfsT-dependent genes and binding sites in the genome of P. aeruginosa P8W after RNA-seq. (A) Volcano plot of the transcriptome data (P8D versus P8W). Significantly differentially expressed genes (DEGs) (log_2_ fold change of ≥1; FDR of ≤0.001) are indicated in red (upregulated) and green (downregulated), while others are represented by gray dots, and genes with potential LfsT binding sites upstream of the start codons are indicated (red triangles and inverted green triangles). (B) Classification of DEGs expressed in response to LfsT deficiency based on PseudoCAP categories. When a gene was assigned to different categories, the first category was selected (see Data Set S3 in the supplemental material). Red and green bars represent the numbers of upregulated and downregulated DEGs, respectively. The PseudoCAP categories were further grouped into six classes (differently colored rectangles on the left). For clarity, some categories with no assigned DEGs are not displayed. (C) Search for the motif NAACN(0,9)GTTN across promoter regions within 500 bp upstream of 113 DEGs. Seventeen genes with predicted sites in the intergenic regions are shown.

Numerous transcriptional regulators with uncharacterized XRE DNA binding domains were found to play critical roles in both secondary metabolism and bacterial adaptation to new environments (20). We conducted a transcriptome analysis to further explore the regulatory role of LfsT in host metabolic processes. As shown in Fig. 5A, the expression levels of a total of 284 genes were significantly altered between P8D and P8W (Data Set S3) (fold change [FC] of ≥2; false discovery rate [FDR] of ≤0.001), including 112 upregulated and 172 downregulated genes. These differentially expressed genes (DEGs) were grouped into 6 major classes (Fig. 5B) based on PseudoCAP (38), where most DEGs belonged to the PE (putative enzyme), AABM (amino acid biosynthesis and metabolism), MP (membrane protein), and AP (adaptation and protection) classes.

Subsequently, we predicted the promoters of these DEGs and found 113 promoters (Data Set S4) within 500 bp upstream of their start codons (39). According to the results in Fig. 2J, we searched for derivative palindromic sequences (5′NAACN(0,9) GTTN-3′) across these potential promoter regions by MAST (Motif Alignment & Search Tool) analysis. Seventeen DEG promoters were identified to contain the putative LfsT binding sites in the intergenic regions, including 8 upregulated and 9 downregulated genes (Fig. 5A). The predicted sites were adjacent to the DEGs, ranging from 14 to 364 bp upstream, and the functions of these 17 DEGs are shown in Fig. 5C. To investigate which genes might be directly regulated by LfsT, we performed EMSAs with the 17 amplified promoter fragments using the indicated primer pairs (Table 2). As shown in Fig. S6, LfsT specifically bound to 4 promoters with different affinities, including *potA* (encoding a polyamine transport protein), *faoA* (encoding a fatty acid oxidation complex alpha subunit), *popN* (encoding a type III secretion outer membrane protein), and *PA2550* (encoding a probable acyl-CoA dehydrogenase). Combined with the results in Fig. 2J, we speculated a more precise binding motif for LfsT, NAACN(5,8)GTTN. These results implied that LfsT possibly functions as a pleiotropic regulator with versatile roles in host metabolism.

### LfsT positively regulates fatty acid degradation.

**FIG 6:**
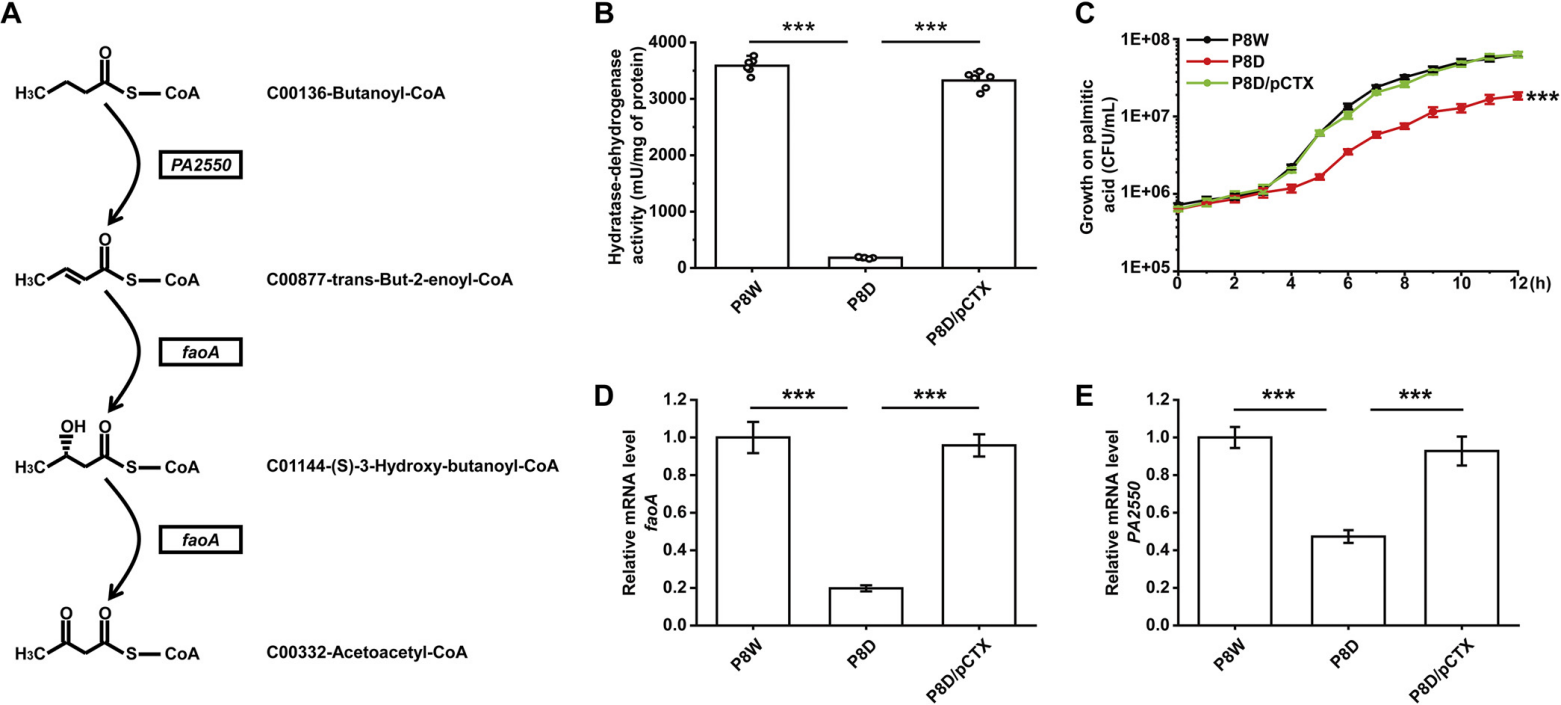
Role of LfsT in fatty acid metabolism. (A) Schematic of the *faoA* (encoding the fatty acid oxidation complex α-subunit) and *PA2550* (encoding a probable acyl-CoA dehydrogenase) genes engaging in the partial dehydrogenation-oxidation of fatty acids. (B) HDT activity assays of the indicated strains. (C) Growth curves of the related strains using 0.1% (wt/vol) palmitic acid as the sole carbon and energy source (supplemented with 100 mg/L ampicillin) for 12 h. (D and E) RT-qPCR assay of *faoA* (encoding the fatty acid oxidation complex α-subunit) and *PA2550* (encoding a probable acyl-CoA dehydrogenase) in the indicated strains at mid-log phase (8 h in LB medium supplemented with 100 mg/L ampicillin). Each sample was tested in triplicate (C to E) or sextuplicate (B), and the experiments were independently replicated three times. Data were analyzed by one-way ANOVA with Tukey’s multiple-comparison test (α < 0.05) to compare the mean differences between the data groups. Error bars show standard deviations. ***, *P < *0.001.

**FIG 7:**
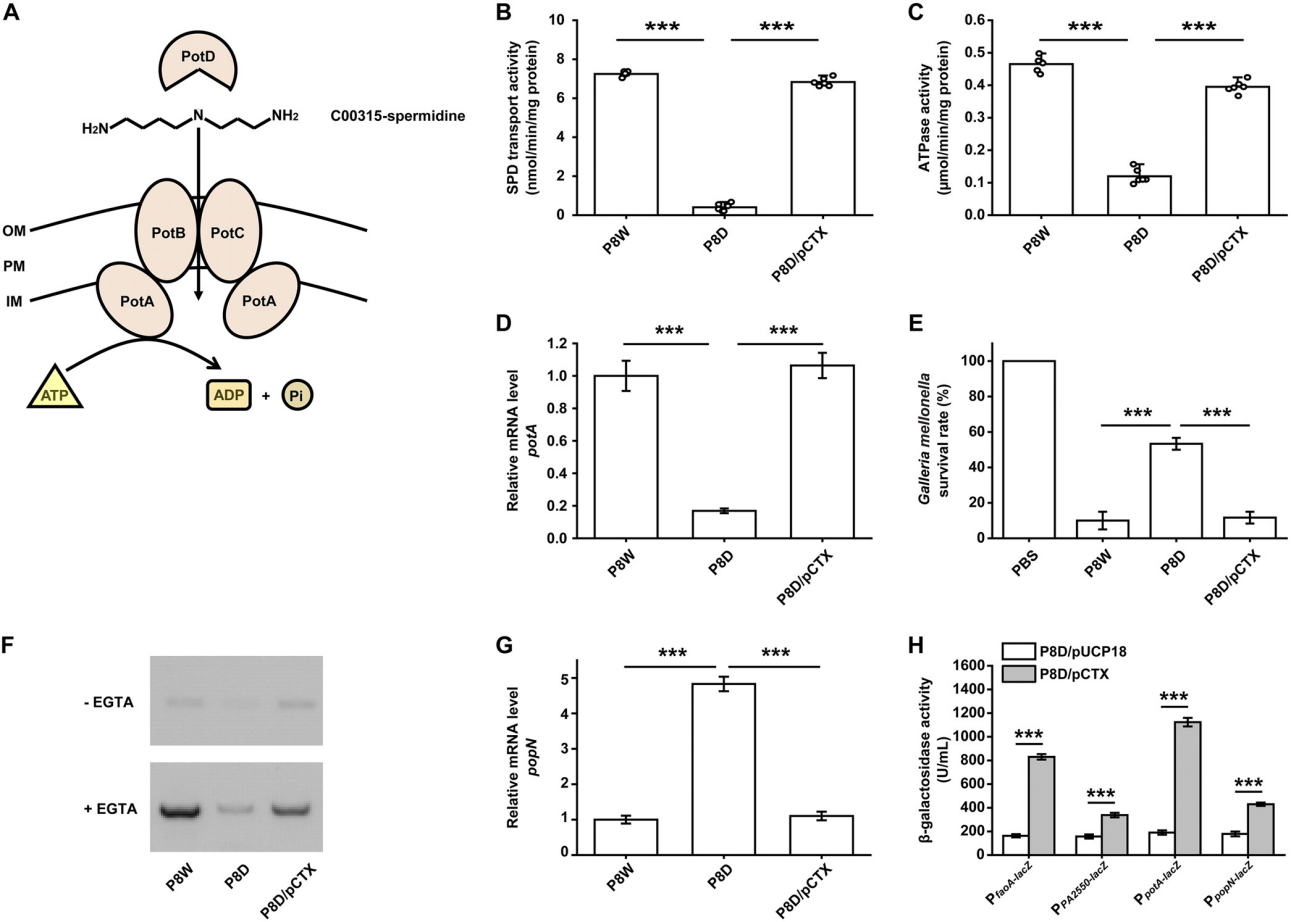
Regulatory functions of LfsT in SPD (spermidine) transport and T3SS activity. (A) Schematic of the SPD transport system comprised of the PotA–PotD proteins in P. aeruginosa. OM, outer membrane; PM, plasma membrane; IM inner membrane. (B) SPD transport activity analysis of the indicated strains. (C) Corresponding ATPase activity assays of the related strains. (D) RT-qPCR assay of the *potA* gene (encoding a polyamine transport protein) in the indicated strains. Details are described above. (E) Galleria mellonella-killing assay using the indicated strains for 24 h. The group treated with phosphate-buffered saline (PBS) is the control group. (F) Detection of secreted ExoS (exoenzyme S) in different bacterial supernatants by Western blotting. (G) RT-qPCR assay for *popN* (encoding a type III secretion outer membrane protein). (H) LacZ activity of the indicated DEG promoter-*lacZ* fusions in P8D carrying pUCP18 or pCTX. The experiments were independently replicated three times, and each sample was tested in triplicate (D to H) or sextuplicate (B and C). Data were analyzed by one-way ANOVA with Tukey’s multiple-comparison test (α < 0.05) to compare the mean differences between the related groups. ***, *P < *0.001. Error bars show standard deviations.

P. aeruginosa grows better aerobically than E. coli on short-, medium-, and long-chain FAs as the sole carbon and energy sources (40). RNA-seq data indicated that the transcription of several FA metabolism-related genes was significantly altered in P8D, including enhanced expression of *fabD*, *fabZ*, and *PA4389* but weakened expression of *adhA*, *faoA*, *PA2550*, and *speA*. Notably, *faoA* (encoding a fatty acid oxidation complex α-subunit) and *PA2550* (encoding a probable acyl-CoA dehydrogenase) are two crucial genes in FA degradation. They function by dehydrogenation-oxidation in the gradual transformation of FAs from long-chain FAs to short-chain FAs, as shown in the schematic in Fig. 6A. A spectrophotometry assay (41) was used to measure HDT (the total multienzyme hydratase-dehydrogenase complex for FA β-oxidation) activity. We found much lower HDT activity in P8D than in P8W and P8D/pCTX, only 5.3% and 5.8%, respectively (*P < *0.001) (Fig. 6B). Furthermore, we used the indicated strains to perform growth experiments on palmitic acid as the sole carbon and energy source. After 12 h of growth, significant growth retardation with less biomass of P8D than of P8W and P8D/pCTX was observed (*P < *0.001) (Fig. 6C). RT-qPCR data for *faoA* and *PA2550* were consistent with the above-described results (*P < *0.001) (Fig. 6D and E). To further identify the role of LfsT in the regulation of *faoA* and *PA2550*, we constructed transcriptional *lacZ* reporter fusions. The *faoA* and *PA2550* promoters were more active in P8D when complemented with the *lfsT* gene than with the empty vector (*P < *0.001) (Fig. 7H). All of these results indicated that LfsT plays a positive role in FA degradation in strain P8W.

### LfsT increases SPD transport and T3SS activity.

Polyamines (putrescine, spermidine, and spermine) are crucial for normal cell growth and viability, having been reported to modulate the functions of RNA, DNA, nucleotide triphosphates, proteins, ion channels, and other acidic substances (42). The polyamine content in cells is dependent on its biosynthesis, degradation, and transport (43). For P. aeruginosa, a spermidine-preferential uptake system comprised of PotA, PotB, PotC, and PotD is schematically depicted in Fig. 7A. The expression levels of *potA*, *potB*, *potC*, *potD*, and *PA5376* were significantly downregulated in P8D, all of which belong to the ATP binding cassette (ABC) transporters in the transcriptome data. The SPD transport activity was determined by measuring [^13^C]spermidine uptake into ornithine-loaded inside-out membrane vesicles. We found that the SPD transport activity in P8D under specific growth conditions (for details, see Materials and Methods) was significantly downregulated compared to those in P8W and P8D/pCTX (*P < *0.001) (Fig. 7B). Besides, we evaluated the ATPase activity of the indicated strains using an ATPase assay kit under the same conditions, and a similar distinct downregulation of the corresponding activity in P8D compared to P8W and P8D/pCTX was observed (*P < *0.001) (Fig. 7C). Data from RT-qPCR and transcriptional *lacZ* reporter fusion analyses of *potA* further supported the above-described results (*P < *0.001) (Fig. 7D and H). These data confirmed the positive impact of LfsT on SPD transport in P8W.

A critical factor in the pathogenesis of acute P. aeruginosa infections is the T3SS, by which the effector proteins are injected directly into host cells (44). Considering the significantly changed expression of T3SS-related genes in P8D (*popN*, *pcr3*, *pcrD*, *pscO*, and *PA1697*), we hypothesized that LfsT might influence P8W virulence *in vivo*. A Galleria mellonella-killing assay using the indicated strains for acute infection (24 h) was conducted. The relative rate of survival of the P8D-infected group was 5.3-fold higher than that of the P8W group, while it 4.6-fold higher than that of the P8D/pCTX group (*P < *0.001) (Fig. 7E). This result was also identified by a Western blot assay. As shown in Fig. 7F, P8D exhibited less ExoS (exoenzyme S) secretion than P8W and P8D/pCTX induced by EGTA. The *popN* gene encodes a T3SS outer membrane protein precursor, which specifically interacts with Pcr1 and forms a T3SS repressor (45). We further determined the negative role of LfsT in regulating *popN* gene expression by RT-qPCR and *lacZ* reporter fusion assays (*P < *0.001) (Fig. 7G and H). These results indicated that LfsT positively regulates T3SS activity in the host bacterium P8W.

## DISCUSSION

Our understanding of bacterium-phage interplays and coevolution is limited, particularly concerning the small regulatory proteins (e.g., XRE-type TRs) in P. aeruginosa due to the lack of a deep understanding of its intricate regulatory systems. In the present study, we report a systematic functional analysis of a putative XRE-type transcriptional regulator, LfsT, in a clinical P. aeruginosa strain isolated from a burn patient, engaging in the control of phage infection and multiple host metabolic processes. These findings highlight an evolutionary mechanism behind bacterium-phage interactions and unveil a full-scale understanding of the regulatory network of P. aeruginosa metabolism and virulence, providing potential clues for novel drug targets designed to handle P. aeruginosa infections.

**FIG 8:**
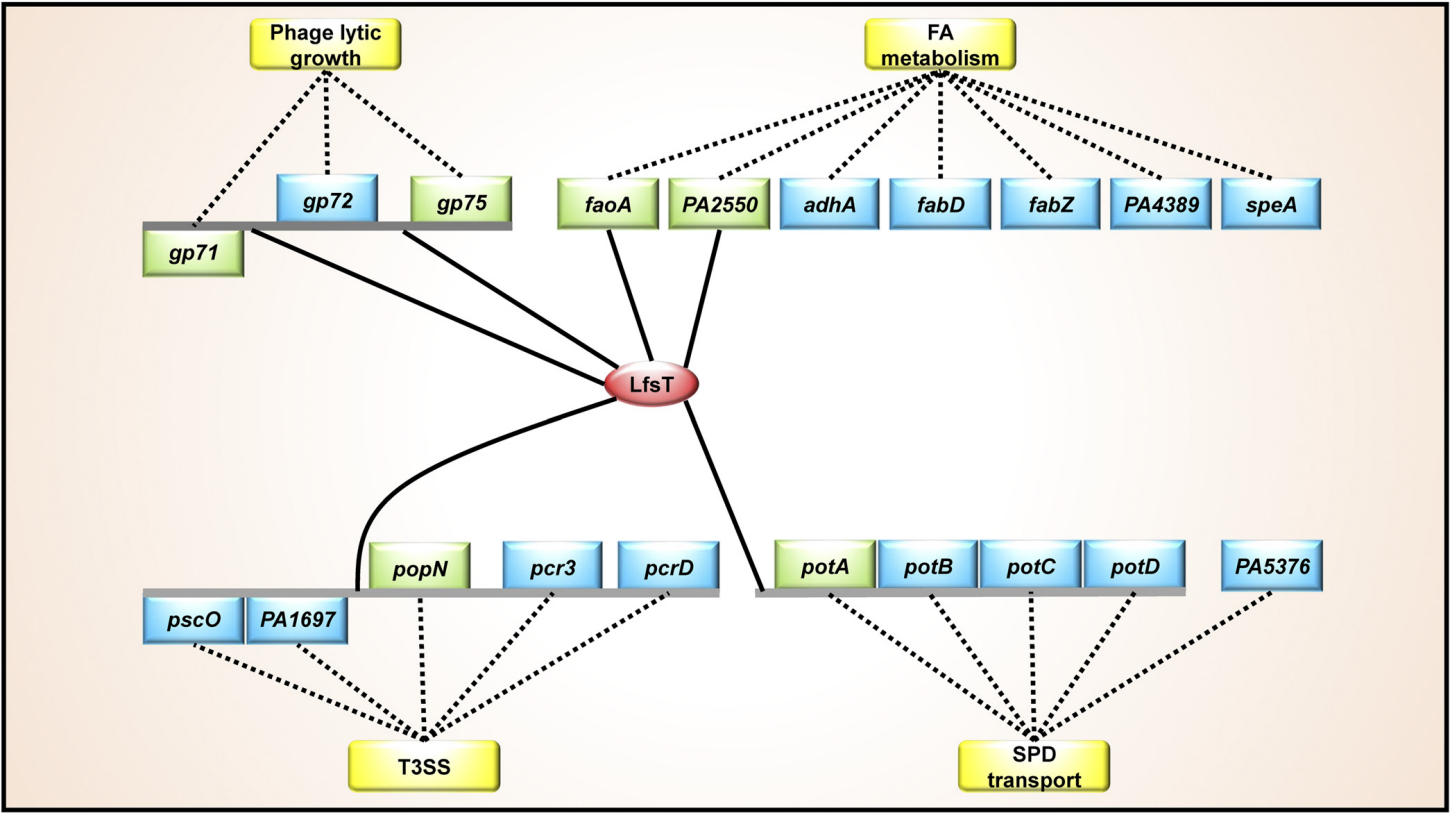
Scheme of the LfsT regulatory network in the host bacterium P. aeruginosa P8W. Solid lines represent LfsT-targeted sites in the promoter regions of individual genes or divergent operons. Dotted lines indicate links (direct or indirect) between the DEGs and various processes (in yellow rectangles) reported previously. The LfsT protein is depicted in the red oval. The genes directly subject to LfsT mediation are depicted in green rectangles, while others involved in the regulation of LfsT are depicted in blue rectangles. FA, fatty acid; SPD, spermidine; T3SS, type III secretion system.

All of the identified functions of LfsT as a novel transcriptional regulator are summarized as a regulatory network in the schematic diagram in Fig. 8. Here, the global regulatory roles of LfsT are involved in several direct targets, including *gp71*, *gp75* (phage infection), *faoA*, *PA2550* (FA degradation), *potA* (SPD transport), and *popN* (T3SS) in P. aeruginosa. LfsT controls the phage sensitivity of a clinical P. aeruginosa strain, similar to the XRE-type regulator SrpA in P. aeruginosa reference strain PAK described previously (46). However, the underlying molecular mechanism of SrpA-mediated phage infection is coregulation with the RNA polymerase of the lytic phage K5 (46), and analogous to the SinR family λ phage repressor, LfsT regulates the transcription of numerous vital phage genes via binding specifically to the promoter regions. The *gp71*/*gp72* gene pair resembles the *ci*/*cro* switch in phage λ, which binds to the same three right operons to perform autoregulation (47). Briefly, at low CI repressor concentrations, O_R_1 and O_R_2 are occupied to ensure that Cro synthesis is repressed while CI synthesis is activated. At high concentrations, O_R_3 is bound, which turns off *ci* gene transcription. The Cro binding pattern is just the opposite (35, 48–50). Interestingly, we found a truncated O_R_3-like operator in the PP9W2 genome by multiple alignments with the corresponding sequences of phage λ, which might be defective and disrupt the binding of the Gp71 and Gp72 proteins at this site. This may partially explain why no difference was found for the transcriptional P*_gp71_*-*lacZ* fusion when complemented with the Gp71 or Gp72 protein. No hysteretic positive band was observed in EMSAs using the *gp72* promoter fragment, indicating the specificity of the binding of LfsT to the predicted motif (Fig. 2J). The binding of LfsT to this site (in green in Fig. S5 in the supplemental material) of the core *gp71* promoter sequences (in yellow) may interfere with RNA polymerase action since it is adjacent to the −10 promoter region (Fig. S5). This is probably why the transcription level of *gp71* was significantly enhanced in P8D (without *lfsT*) during the latent period. Besides, the O_R3_-like operator overlaps the putative LfsT binding site, which further indicates the negative impact of LfsT on *gp71* gene transcription.

In addition, the negative regulatory role of excess LfsT in some phage gene promoter activities (Fig. 3A and B) may be ascribed to DNA bending caused by the created multimers responsible for the repression state of TRs (51). Moreover, LfsT significantly represses the expression of some lysogeny-related genes and the lysogeny frequency of PP9W2 (Fig. 4). We speculate that the *gp71*/*gp72* switch in phage PP9W2 is probably defective and disabled to fine-tune phage lysis-lysogeny decisions. The binding of LfsT to the predicted site in the *gp71* promoter may remediate the defective regulation, directly limiting the predisposition to lysogeny and indirectly promoting the lytic development of the phage. The *lfsT* gene was possibly acquired via horizontal gene transfer (HGT) since its genomic context is between two prophage regions comprising numerous conjugal transfer-related genes (Fig. S3A). The role of LfsT in phage infection may develop to be an autoregulatory tool for specific phages for better adaption to undesirable genetic mutations under evolutionary pressure.

A more specific motif preferentially recognized by LfsT was identified as NAACN(5,8)GTTN using the other four predicted DEG promoter binding sites. It resembles a partial palindromic structure with a flexible center characterized by AT-rich regions at both ends, which is consistent with most transcriptional regulator binding sites (52). Furthermore, we searched for the motif across the genome of P8W in the Pseudomonas Genome Database, and more than 300 potential LfsT binding sites were identified (data not shown), indicating the possible broad LfsT protein interactions with DNA. Our data suggested that LfsT binding is not limited to one target. It may be speculated that LfsT maintains preferred nucleotide positions with a variable center in recognized sequences under evolutionary stress to exert a global regulation effect on host gene expression.

On the other hand, LfsT also significantly impacts versatile host metabolic processes to different extents. We found that the Δ*lfsT* mutant displayed worse HDT activity and adaptation to the growth of cells on palmitic acid as the sole carbon and energy source. This may be attributed to the downregulation of *faoA* and *PA2550*, which were directly subjected to LfsT-mediated regulation. Transcriptome analysis indicated another factor responsible for regulating FA metabolism, probably involving LfsT-dependent changes in DEGs such as *adhA* (alcohol dehydrogenase), *fabD* (malonyl-CoA-acyl carrier protein transacylase), *fabZ* [(3R)-hydroxymyristoyl-acyl carrier protein dehydratase], *PA4389* (3-oxacyl-acyl carrier protein reductase), and *speA* (arginine decarboxylase), which have been reported to be associated with the biosynthesis, metabolism, and degradation of FA (53–57). It is worth mentioning that LfsT may regulate the transcription of divergent operons rather than individual genes (Fig. 8). The *potA*–*potD* operon is subject to LfsT regulation since the transcription levels of all of these genes were significantly diminished in the absence of LfsT. The expression of the PotA–PotD proteins is required for high SPD transport activity (43, 58–60). The *lfsT*-deficient mutant exhibited decreased SPD and ATPase activity compared to the parent strain, indicating stimulation by LfsT binding to the *potA*–*potD* promoter. We speculate that the interaction of LfsT with DNA may encompass core promoter sequences, positively influencing RNA polymerase activity and resulting in the promotion of *potA*–*potD* gene expression. Besides, *lfsT* is not an essential gene for normal cell growth in LB medium (Fig. 1A). However, it impacts SPD uptake and ATPase activity tested in medium A (see details in Materials and Methods), indicating that the regulation of the *potA*–*potD* operon by LfsT may depend on specific growth conditions. Traditionally, the development of antimicrobials for anti-P. aeruginosa therapeutics is based on interference with essential gene targets (61). Our findings expand the useful weapons of “nonessential genes” (e.g., the *lfsT* gene acquired by HGT) for potential targets for the development of novel drugs against P. aeruginosa infections.

In P. aeruginosa, FA and SPD have been reported to regulate T3SS expression and virulence *in vivo* by modulating intercellular signal molecules (62–64). Particularly, significantly increased expression levels of the *pscO*-*PA1697* and *popN*-*pcrD* genes were found in response to LfsT deficiency. LfsT binding to the predicted site from bp 66 to 88 upstream of *popN* was identified, which might reduce the access of RNA polymerase to −10 promoter sequences. We observed an increased rate of Galleria mellonella survival in the absence of LfsT. This might be manifested by the decreased secretion of ExoS (Fig. 7E and F). Our results suggested the role of LfsT in *popN*-*pcrD* operon inhibition, and we speculate that it is an energy-efficient way for these genes to be repressed in a tightly controlled manner in the cell to save resources for gene expression. Taken together, these results show that the acquired LfsT, probably by HGT, has evolved into an important functional regulatory protein for host bacterial metabolism and virulence.

In light of the global regulatory effects of LfsT, we performed a DIAMOND BLASTP search of the Pseudomonas Genome Database with default parameters. A total of 134 complete genomes contained LfsT homologs (Data Set S5), about 20% of the total complete genomes online, indicating that LfsT-like proteins are widely distributed among Pseudomonas strains. However, its performance in various strains may differ due to the discrepant genomic contexts. It is not surprising that some previously described genes exert different regulatory effects on various bacterial subspecies (15, 65, 66). We speculate that this may be due to many factors, e.g., the existence of an alternative pathway in the complicated transcriptional regulatory web, which allows the complex and precise response of bacterial cells to environmental stress. This study adds a new player (LfsT) to the existing network of multiple metabolic pathways in P. aeruginosa, providing a deeper understanding of phage-host interactions and indicating the role of these XRE-type TRs in the adaptation of host cells to rapidly changing environments. However, the precise role of LfsT in P. aeruginosa awaits elucidation; e.g., (i) analogous to other XRE-type TRs, LfsT regulation probably requires other specific signaling molecules or growth conditions apart from binding sequences; (ii) LfsT improves the metabolic activity of the bacteria, while it also increases the phage sensitivity of the host, which may be speculated by adaptive costs under continual coevolution between bacteria and phages; (iii) some LfsT-dependent targets are also involved in another intricate regulatory network that may be coregulated by other TRs; and (iv) how these LfsT targets are expressed in response to excess LfsT remains to be determined. Further studies are required to determine the underlying molecular mechanisms engaged in these ways.

## MATERIALS AND METHODS

### Bacterial strains, phages, plasmids, and culture conditions.

The bacterial strains, phages, and plasmids used in this study are detailed in Table 1. We routinely cultured the strains in LB medium supplemented with the appropriate antibiotics at 37°C unless stated otherwise. The primers utilized in this work are detailed in Table 2.

### Characterization of phage PP9W2.

We performed a phylogenetic analysis based on the major capsid proteins of the selected D3-like Pseudomonas phages. The neighbor-joining tree (topology only) was constructed using MEGA 7, and the branch lengths are shown in Fig. S1B in the supplemental material. A growth inhibition experiment was conducted as previously described (67). Briefly, the indicator strain P8W was incubated with phage PP9W2 at different MOIs (0.1, 1, and 10). Next, the mixtures were added to fresh LB medium for 12 h of growth. The optical densities of the samples were measured in triplicate at 1-h intervals. A one-step growth experiment was performed as previously described (68). In brief, the medium of a P8W culture grown overnight was mixed with a purified phage PP9W2 solution (MOI = 1) supplemented with 10 mM CaCl_2_. The culture was continuously incubated at 37°C for 6 h. We obtained samples (100 μL) in triplicate every 30 min and determined the phage titer by an overlay agar plate method.

### Construction of a Tn*5G* mutant library.

We used transposon Tn*5G* to generate a mutant bank to screen for phage PP9W2-resistant mutants of strain P8W as described previously, with minor modifications (69). Briefly, the bank was cultivated at 37°C for 2 h and then incubated with the purified phage PP9W2 stock solution. The mixture was plated onto Luria-Bertan (LB) medium-agar plates with 100 μg/mL ampicillin and gentamicin. A spotting assay was used to identify the phage-resistant mutants.

### Identification of insertion sites.

Inverse PCR was performed to identify the insertion sites in the PR mutants as described previously (70). The PCR fragments amplified by using the primer pairs listed in Table 2 were cloned into the pGEM T-Easy vector for further sequencing. The genome of P8W was used to analyze sequence homology.

### LPS profile and content analyses.

LPS profile analysis of the related strains and mutants was performed as previously described (71). We used an LPS extraction kit (catalog number ab239718; Abcam) to extract LPS. The total carbohydrate content (grams per CFU) was quantified according to a total carbohydrate quantification assay (catalog number ab155891; Abcam).

### Adsorption rate assay.

Briefly, the related cells cultured overnight were resuspended in fresh LB medium supplemented with 10 mM CaCl_2_; next, the culture was mixed with the purified phage stock solution (MOI = 1) and incubated without shaking at 37°C for 30 min. The phage adsorption rate was quantified as previously described (72).

### Construction of the *lfsT* gene deletion derivative P8D.

We used a homologous-recombination method to knock out the *lfsT* gene in the P8W genome. Briefly, we first amplified the upstream and downstream fragments of the *lfsT* gene and ligated the molecules. Next, the fragment was ligated with the enzyme-digested plasmid pEX18Tc. The resulting plasmid, pEX18Tc::UD, was first introduced into E. coli DH5α, then transformed into E. coli S17-1, and finally introduced into P8W by conjugation. The *lfsT* gene mutants were screened using L-agar plates supplemented with 100 mg/L tetracycline, 50 mg/L kanamycin, and 7.5% sucrose. The positive clones were identified by PCR and DNA sequencing.

### Complementation experiment.

The amplicon of the *lfsT* gene was digested with the corresponding restriction enzymes and cloned into plasmid pUCP18. The recombinant plasmid (pCTX) was transformed into competent cells of P8D by electroporation. The complemented clone was screened using L-agar plates supplemented with 100 mg/L ampicillin and further identified by PCR and DNA sequencing.

### Growth curve experiment.

Strains cultured overnight were inoculated into fresh LB medium on 96-well plates. The optical density at 600 nm (OD_600_) value was quantified by using an automatic microplate reader (Bioscreen, Finland) at 5-min intervals for 20 h of growth. The test was independently replicated three times, and each sample was tested in quintuplicate.

### LfsT protein structure prediction.

Domain prediction was performed using an online tool (http://smart.embl-heidelberg.de/). We searched the amino acid sequence of LfsT across the nonredundant (nr) database by PSI-BLAST with an expected threshold of 1E−10. A total of 1,000 LfsT-homologous sequences (Data Set S1) were used to generate a WebLogo figure using an online tool (http://weblogo.threeplusone.com/create.cgi). SWISS-MODEL was used to conduct homology modeling of the LfsT-like protein structure as previously described (73). Another 1,000 LfsT homologs (Data Set S2) were involved in the analysis. Sixty-four residues (66% of the LfsT sequence) were modeled with 99.5% confidence by the single highest-scoring template, d1b0na2 (SinR domain-like). Ninety residues (93%) could be modeled at >90% confidence using multiple templates.

### Analysis of the packaging process for phage PP9W2 genomic DNA.

The manipulation suite (Table S1) of the phage PP9W2 genomic DNA sequence was analyzed as described previously (74). Purified phage genomic DNA was digested with the restriction enzyme FseI and then, immediately or after heating (75°C for 10 min), separated on a 0.8% agarose gel by electrophoresis.

### Southern blot analysis.

Briefly, the related strains were incubated with phage PP9W2 at an MOI of 1 according to the growth inhibition experiment results. We sampled the strains at 0 min (control), 15 min (after adsorption), 75 min (1 h of infection), and 135 min (2 h of infection). Total DNA was extracted from the collected cells and then digested with the restriction endonuclease FseI. The primer pairs listed in Table 2 were used to synthesize the designed probe region of *gp02* from bp 1304 to 1805 of the PP9W2 genome to detect the genomic DNA of phage PP9W2 (Fig. S4B). The genomic DNAs of P8W and phage PP9W2 were selected as negative and positive controls, respectively. Probe synthesis and DNA hybridization were performed using detection starter kit II (Roche), according to the manufacturer’s instructions.

### Reverse transcription-quantitative real-time PCR.

We incubated strain P8W, P8D, or P8D/pCTX with phage PP9W2 at an MOI of 1. We sampled the culture every 15 min or 30 min and immediately performed total RNA extraction using a total RNA extraction kit (Promega). Next, 1 μg total RNA was subjected to reverse transcription using the FastKing gDNA dispelling RT supermix (TianGen). The cDNA was finally subjected to RT-qPCR analysis with the primers indicated in Table 2. A total volume of 20 μL comprising 10 μL of TB green premix ExTaq II (TaKaRa Bio), 8.2 μL of deionized water, 0.4 μL each of the primers (10 mM), and 1 μL of the template was used for analysis in triplicate. A typical 2^−ΔΔ^*^CT^* method was used to perform relative quantification (75).

### Electrophoretic mobility shift assay.

We first purified the LfsT protein. Plasmid pET32a(+) was used to generate a recombinant plasmid, pOEX, for the expression of the *lfsT* gene in E. coli BL21(DE3).

A total of 1.5 mM isopropyl β-d-1-thiogalactopyranoside (IPTG) was added to induce the overexpression of *lfsT*. We used a one-step bacterial active protein extraction kit (Sangon Biotech) and a Ni-nitrilotriacetic acid (NTA)-Sefinose column (Bio Basic, Canada) to extract the His-tagged LfsT protein according to the manufacturers’ protocols. A desalting gravity column (Sephadex) was used to purify the target protein.

For electrophoretic mobility shift assays (EMSAs), we amplified the predicted promoter sequences (Table S2 and Data Set S4) of the tested genes with the primers indicated in Table 2. The PCR fragments (150 to 250 bp) were first cloned into pMD19 (Simple; TaKaRa), and the ligated fragments were then further amplified using the primer pair M13-47 and RV-M. We used an EMSA kit (Molecular Probes) to perform the analysis according to the manufacturer’s instructions. The DNA-protein complexes were separated by 5% nondenaturing polyacrylamide gel electrophoresis. The gel was visualized with an automatic digital gel image analysis system (Tanon 1600R).

### Construction of *lacZ* transcriptional reporters.

The predicted promoter fragments of the tested genes were amplified with the primers indicated in Table 2. The related PCR products were cloned into the digested pDN19lacΩ plasmid (76). The recombinant plasmids were then transformed into different strains by electroporation. The activities of the corresponding promoters were represented by the β-galactosidase activities of the double transformants.

### Lysogeny frequency assay.

The lysogeny frequency of phage PP9W2 in different strains was quantified by a modified quantitative PCR method as previously described (77). Briefly, the number of total P8W copies was quantified based on the single-copy gene *PA1789*. The number of lysogen copies was quantified using the primers indicated in Table 2, which amplified the fragment only when the phage was integrated. We sampled different strains after 2 h or 12 h of growth to quantify the lysogeny frequency.

### Transcriptome analysis of P8D compared to P8W.

The related bacterial cells were grown to an OD_600_ of 0.8, and the harvested cells were immediately subjected to freezing in liquid nitrogen and delivered to Novogene (Beijing, China) on dry ice. Briefly, total RNA was extracted and subjected to rRNA removal; purified mRNA was fragmented before cDNA synthesis. The cDNA libraries were sequenced in triplicate on an Illumina HiSeq platform. Alignment of the clean reads to the reference genome (P. aeruginosa P8W [GenBank accession number NZ_CP081477.2]) was conducted using Bowtie (78). A total of 17,737,358 (97.37%) reads were mapped to the reference genome in the sample of P8W, and 19,416,320 (95.67%) reads were mapped to the reference genome in the sample of P8D. Genes with an adjusted *P* value of <0.05 by DESeq were assigned as differentially expressed, of which the DEGs with an FDR of ≤0.001 and a fold change (FC) of ≥2 were regarded as significantly changed. The Pseudomonas Genome Database (https://www.pseudomonas.com/) was used to assign the DEGs based on PseudoCAP. P. aeruginosa PAO1 was selected as the reference genome to convert gene identifiers (http://kobas.cbi.pku.edu.cn/kobas3/annotate/) for comparison.

### HDT activity assay.

We measured the overall activities of enoyl-CoA hydratase and 3-hydroxyacyl-CoA dehydrogenase (HDT) as described previously (41). In brief, the related bacterial cells cultured overnight were harvested and resuspended in 1 mL of Tris-EDTA (TE) containing 0.5% (wt/vol) Triton X-100, 50 μg/mL of DNase I and RNase A, and 1 mg/mL of lysozyme. After 15 min at 37°C, the supernatants were collected and used as the cell extracts. The protein concentrations in the cell extracts were determined by BSA (bovine serum albumin) quantitative method. Next, we mixed 100-μL cell extracts with an equal volume of a reaction mixture comprised of 80 mM Tris-HCl (pH 8.0), 4 mM CoASH (coenzyme A, SH: sulfydryl), 2.5 mM 4-hydroxybenzoic acid and 4-aminoantipyrine, 0.6 mM 3-oxohexadecanoyl-CoA, 0.125% (wt/vol) Triton X-100, 0.5 mg/mL bovine serum albumin (BSA), acyl-CoA oxidase (12 U/mL), and peroxidase (12.5 U/mL). After 5 min at 37°C, 0.5% (wt/vol) SDS was added to terminate the reaction, and the corresponding absorption at 500 nm was then measured. HDT activity was calculated as described in reference (41).

### Growth experiments on palmitic acid.

We cultured the related strains using 0.1% (wt/vol) palmitic acid as the sole carbon and energy source for 12 h. We sampled the cultures every hour and measured the OD_600_ value. The experiment was independently replicated three times, and each sample was tested in triplicate.

### SPD transport activity analysis.

We measured spermidine uptake activity as previously described (43), with minor modifications. Briefly, the related bacterial cells were cultured in medium A containing 0.4% (wt/vol) glucose, 0.7% (wt/vol) K_2_HPO_4_, 0.3% (wt/vol) KH_2_PO_4_, 0.05% (wt/vol) sodium citrate, 0.1% (wt/vol) (NH_4_)_2_SO_4_, 0.01% (wt/vol) MgSO_4_ · 7H_2_O, 0.0002% (wt/vol) thiamine, 0.001% (wt/vol) biotin, and 0.01% (wt/vol) each of leucine, threonine, methionine, serine, glycine, and ornithine supplemented with 100 mg/L ampicillin at 37°C until the OD_600_ reached 0.3. Next, cells were harvested and resuspended in buffer I [0.4% glucose, 62 mM KH_2_PO_4_ (pH 7.0), 1.7 mM sodium citrate, 7.6 mM (NH4)_2_SO_4_, and 0.41 mM MgSO_4_] to generate a final protein concentration of 0.1 mg/mL. Next, the suspension was mixed with 0.25 mM [^13^C]spermidine and incubated at 30°C for 5 min. The cells were collected on cellulose acetate membrane filters, and the filters were washed three times with buffer I. δ^13^C values were measured using an isotope ratio mass spectrometer (Picarro analyzer).

### ATPase activity assay.

We cultured the related strains under the same conditions as the ones described above for the SPD transport experiment. An ATPase assay kit (colorimetric, catalog number ab234055; Abcam) was used to monitor ATPase activity in the cell extracts.

### Galleria mellonella-killing assay.

A Galleria mellonella-killing assay was performed as previously described (79). Briefly, strains cultured overnight were diluted (1:100) into fresh LB medium at 37°C until the OD_600_ reached 0.5. Next, the cells were collected, resuspended to a final concentration of 1 × 10^5^ CFU/mL (plate count), and supplemented with 10 mM MgSO_4_. Ten microliters of the diluted strain solution or MgSO_4_ (aqueous) was injected into Galleria mellonella larvae and incubated at 37°C for 24 h. Each group comprised 20 larvae and 3 replicates. The experiments were independently replicated three times.

### Western blot analysis.

We performed Western blotting as previously described (80). Strains cultured overnight were resuspended (1:100) in fresh LB medium supplemented with 5 mM EGTA or double-distilled water (ddH_2_O) and further incubated at 37°C for 4 h. The supernatants were harvested and mixed with 15% trichloroacetic acid (TCA). The samples (equal bacteria loads, with 500 μL of NuPAGE antioxidant) were run on 12% NuPAGE Bis-Tris gels (Thermo Fisher Scientific). A nitrocellulose membrane was used to transfer the protein. Rabbit antiserum (1:1,000) was used to detect ExoS. Hybridization was performed using 1 mg/mL secondary antibody (1:2,000), and the membrane was then incubated with Amersham ECL Prime Western blotting detection reagent (Thermo Fisher Scientific).

### Data availability.

The whole-genome sequencing data of P8W were deposited at the Sequence Read Archive (SRA) of the National Center for Biotechnology Information (NCBI) under BioProject accession number PRJNA753640. The whole-genome sequencing data of PP9W2 have been made available at the NCBI under GenBank accession number OM141125. The RNA-seq data have been deposited at the Sequence Read Archive of the NCBI under BioProject accession number PRJNA857324. Other related data are available in this article and the supplemental material or from the corresponding authors upon request.
