# Supplementary material for: A Novel XRE-Type Regulator Mediates Phage Lytic Development and Multiple Host Metabolic Processes in Pseudomonas aeruginosa
Source: Microbiol Spectr. 2022 Nov 29;10(6):e03511-22. doi: 10.1128/spectrum.03511-22 (PMC9769523; doi:10.1128/spectrum.03511-22)
Supplement: Supplemental file 1 — Supplemental material. Download spectrum.03511-22-s0001.pdf, PDF file, 0.8 MB [file spectrum.03511-22-s0001.pdf]

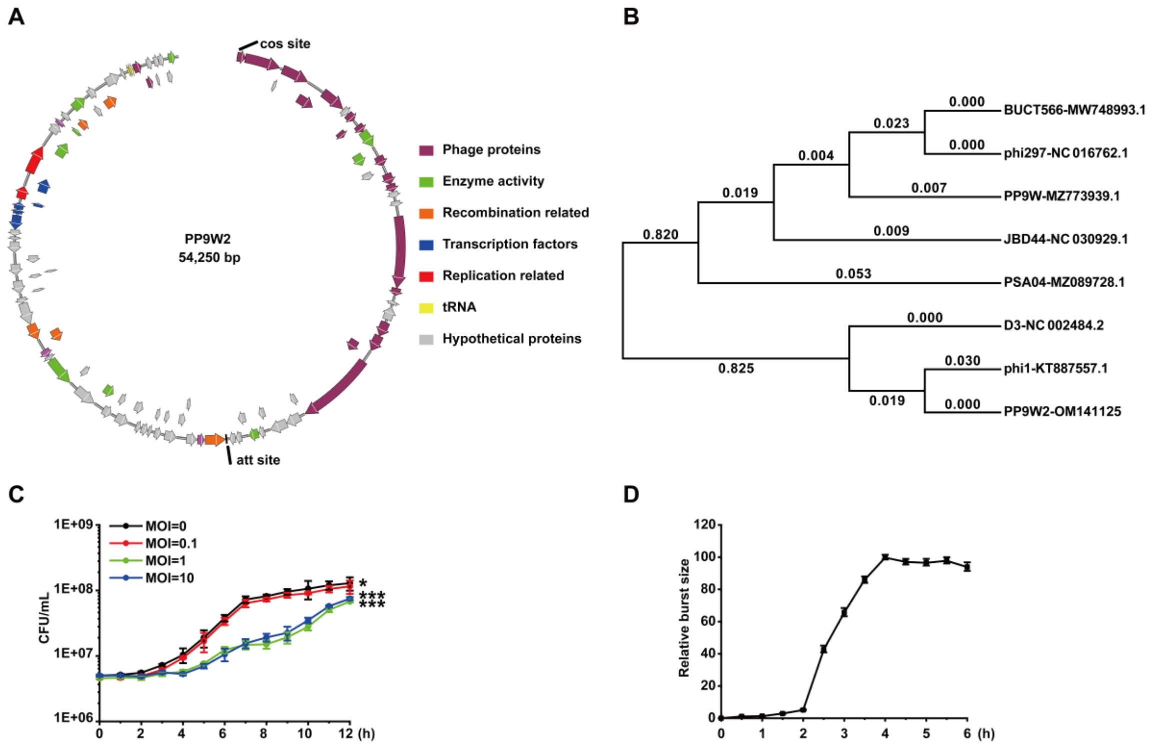

Fig S1. Characterization of the phage PP9W2. (A) The genome of PP9W2 is linear double-stranded DNA around 54-kb. It has a 12-bp cos site similar to *Pseudomonas* phage D3, and a 61-bp attachment (att) site engaging in integrating into the genome of the *P. aeruginosa* strain P8W. Different colored rectangles represent various proteins. (B) The phylogenetic tree (topology only) based on the major capsid proteins of the indicated D3-like *Pseudomonas* phages. Branch lengths are displayed by the numbers. (C) Growth inhibition experiment of the phage PP9W2 on the strain P8W at different MOI (multiplicity of infection, the initial dose ratio of PP9W2/P8W). (D) One-step growth experiment of the phage PP9W2 indicated by the strain P8W (MOI=1). The experiments were independently replicated three times and each sample was tested in triplicate (C-D). Data were analyzed by one-way analysis of variance (ANOVA) with Tukey's multiple comparisons ($\alpha < 0.05$) to examine the mean

differences between the end point of data groups. *, $P < 0.05$. ***, $P < 0.001$.

Error bars show standard deviations.

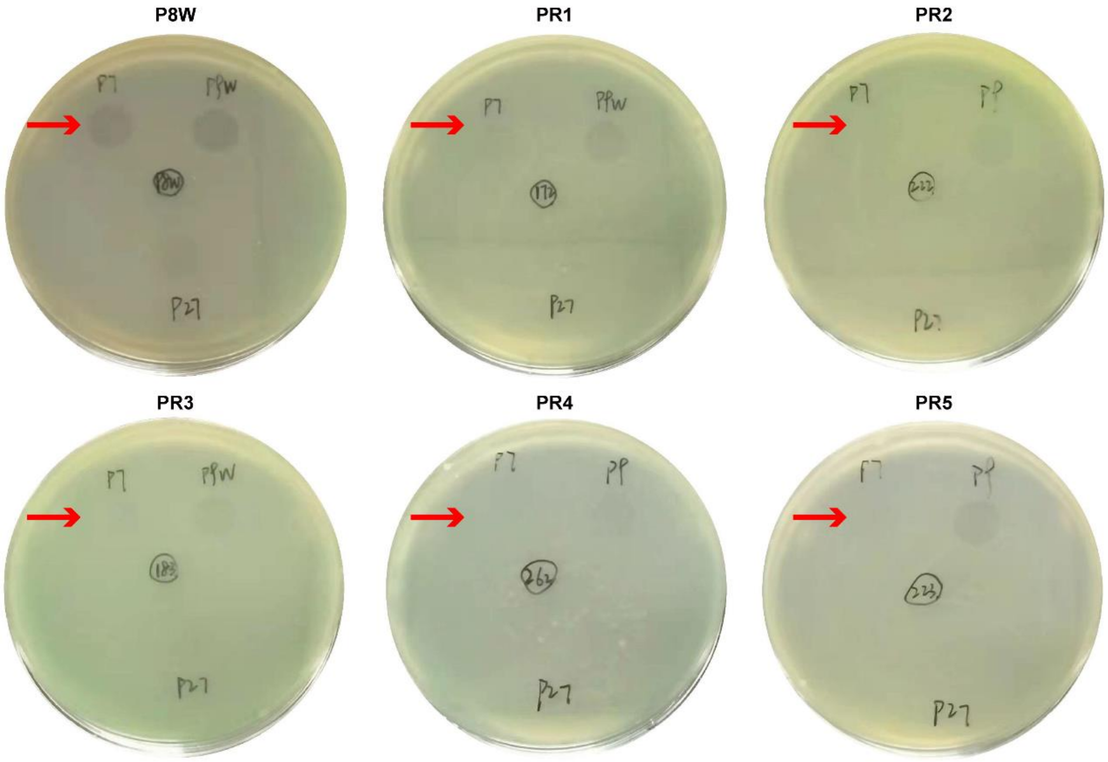

Fig S2. Phage-resistant (PR) mutants of P8W. A spotting test was used to detect the mutants' sensitivity to different phages. Red arrows show the spotting sites of the phage PP9W2 on the indicated plates. The other spotting sites represent the phages not related with this study.

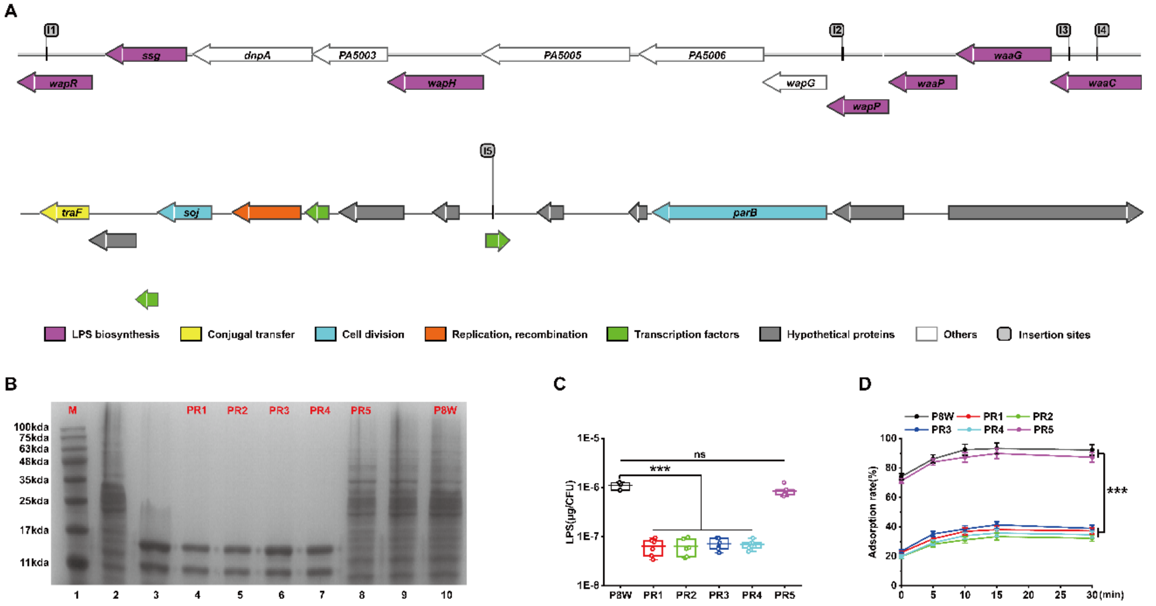

FIG S3 Identification of the Tn*5G* inserted sites and characterization of the PR mutants. (A) Different inserted sites (I1-5) in the genome of P8W. I1-4 were inserted in a cluster of genes involved in LPS synthesis, including *wapR* (encodes the alpha-1,3-rhamnosyltransferase), *wapP*, and *waaC* (encodes the heptosyltransferase I). I5 was inserted right behind 81st bp of a gene (WP_006379425.1) that encodes a putative XRE family transcriptional regulator. (B) LPS profile analysis. LPS structure was assessed by SDS–PAGE (15% gel). Lanes 1, a protein marker; Lanes 4-7, PR1-4; Lane 8, PR5; Lane 10, LPS control from P8W. The other lanes are irrelevant with this study. (C) LPS content assay of the indicated strains. (D) Adsorption rate analysis of the related strains infected with the phage PP9W2 at MOI of 1. Each sample was tested in triplicate (C-D) and the experiments were independently replicated three times. Data were analyzed by one-way analysis of variance (ANOVA) with Tukey’s multiple

comparisons (α < 0.05) to compare the mean differences between the data groups. Error bars show standard deviations. ***, $P < 0.001$.

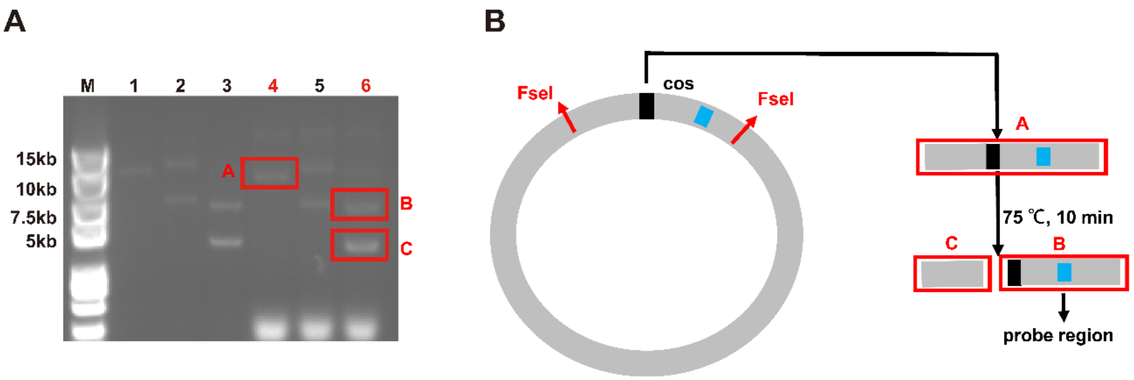

Fig S4. Phage packing analysis. (A) M: DNA marker; Lane 4: the purified phage PP9W2 genomic DNA digested with FseI running on 0.8% agarose gel electrophoresis. Lane 6: after heating (75 ℃, 10 min), corresponding to Lane 4. The other Lanes are not related with this study. (B) Schematic representation of the enzyme FseI digesting process. Red box A represents a fragment from the end junction of the concatemeric genomic DNA. Red box B represents a fragment from the 5′ end of the monomeric genomic DNA with a designed probe region (in blue). Red box C represents a fragment from the 3′ end of the monomeric genomic DNA.

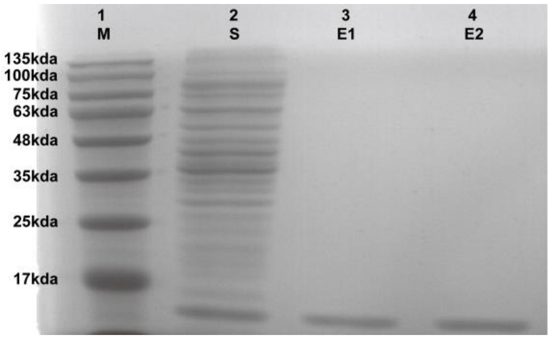

Fig S5. SDS-PAGE (5% concentrated gel, 15% separated gel) results of the purified His-tagged LfsT protein. Lane 1: the protein marker; Lane 2: the supernatant of lysed cells by One Step Bacterial Active Protein Extraction Kit; Lane 3: the first eluent of the His-tagged LfsT protein; Lane 4: the second eluent of the His-tagged LfsT protein.

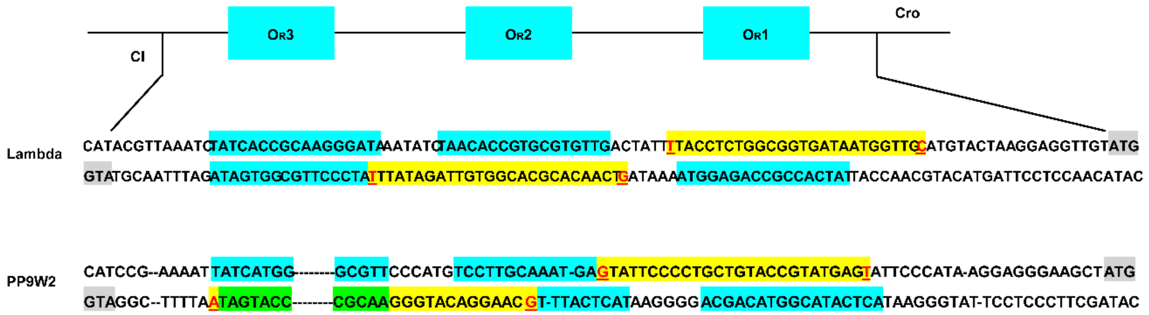

Fig S6. Comparing the intergenic region sequence between *gp71*/*gp72* to the CI/Cro switch in the lambda phage. OR1-3 are three right operators (blue boxes, upper panel). The corresponding sequence are extended in the middle panel. The start codons are indicated in grey, the (-10, -35) core promoter regions (in the middle of the red letters) are shown in yellow. For convenience, we use "-" represents the gaps in multiple alignments. We found a truncated OR3-like operator (lower panel) adjacent to the start codon of the *gp71* gene. The predicted LfsT binding site is indicated in green, which also overlaps the OR3-like operator within the core promoter region of *gp71*.

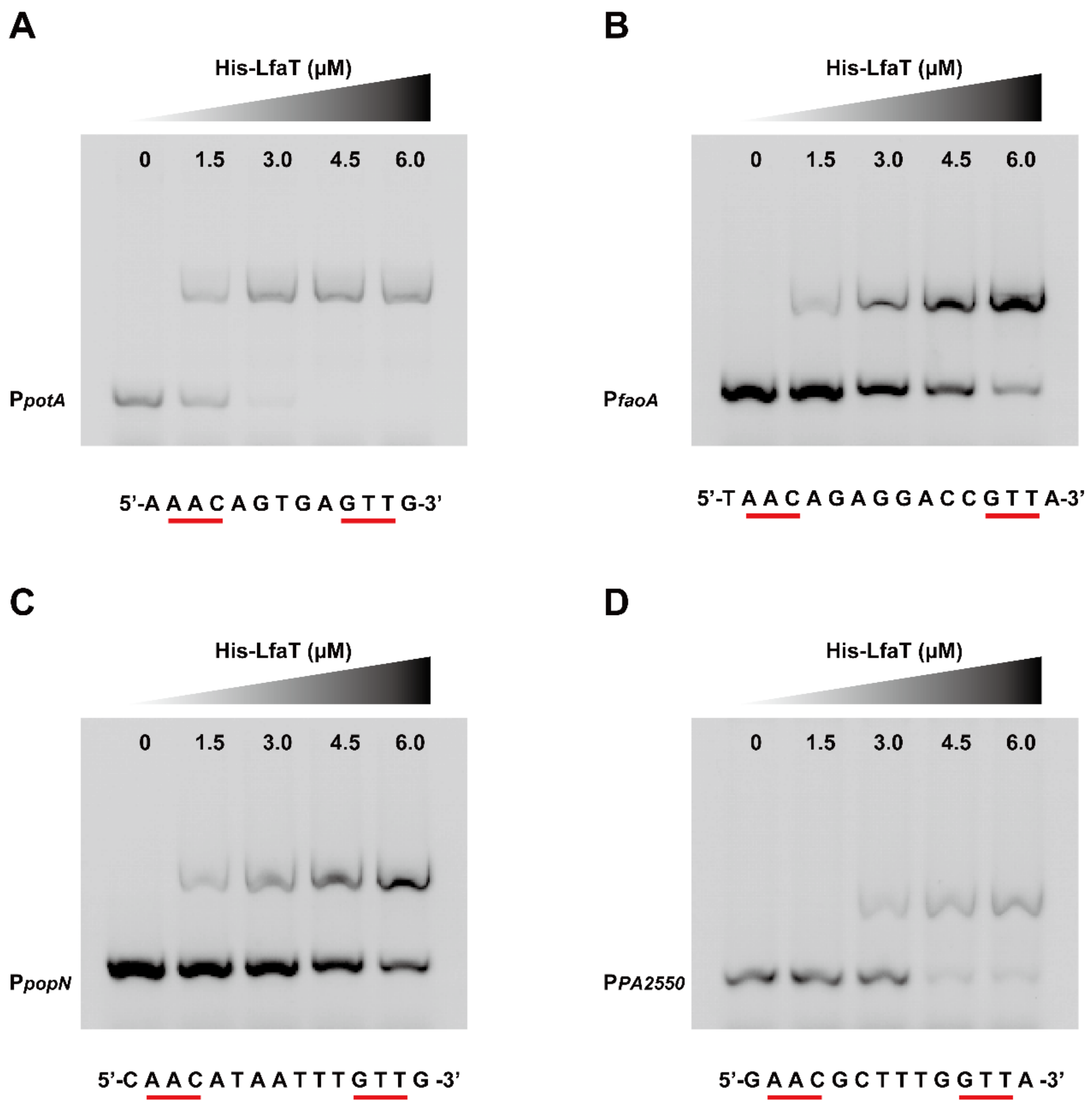

Fig S7. EMSA analysis of the LfsT binding sites in the promoter regions of four DEGs. The palindromic sequences of each binding site are underlined in red.

Table S1. Restriction summary results of the PP9W2 genome.

Enzyme digestion sites:	Positions (bp)
AflII c|ttaag	18803, 32150
AvrII c|ctagg	29177
BamHI g|gatcc	20545, 53170
BfrI c|ttaag	18803, 32150
BlnI c|ctagg	29177
FseI ggccgg|cc	7701, 48702
HpaI gtt|aac	42326, 52849
PsiI tta|taa	8282, 11006
SnaBI tac|gta	48901, 49661
XbaI t|ctaga	22522, 41099

Table S2. Promoter sequences of six phage genes bound by LfsT.

Gene	Promoter Sequences（50bp）	Upstream from the start codons (bp)
gp75	acgcttggggaaacacaacacacaggaagcataacccat gagttacgggt	-37
gp71	catttgcaaggacatgggaacgcccatgataattttcggatg gaactcaa	-39
gp13	atcaggctgactgcctgtcctccaagcactacaagccgggtg agcatatc	-177
gp09	tctttggccatagccaggggcgccaagcgaatcatccttctcg gctacga	-244
gp04	tggcatgacgggaacccagtcatgacctggatgatgggcaa cgtggcggc	-375
gp01	gttttcactggactcgtgcggcttgacagaaaaatctagttaa atgagaa	-154
